# Exploring and expanding the Fe-terephthalate metal–organic framework phase space by coordination and oxidation modulation†

**DOI:** 10.1039/d1mh01663f

**Published:** 2021-10-13

**Authors:** Dominic Bara, Emily G. Meekel, Ignas Pakamorė, Claire Wilson, Sanliang Ling, Ross S. Forgan

**Affiliations:** WestCHEM School of Chemistry, University of Glasgow, Joseph Black Building, University Avenue Glasgow G12 8QQ UK Ross.Forgan@glasgow.ac.uk; Advanced Materials Research Group, Faculty of Engineering, University of Nottingham University Park Nottingham NG7 2RD UK

## Abstract

The synthesis of phase pure metal–organic frameworks (MOFs) – network solids of metal clusters connected by organic linkers – is often complicated by the possibility of forming multiple diverse phases from one metal–ligand combination. For example, there are at least six Fe-terephthalate MOFs reported to date, with many examples in the literature of erroneous assignment of phase based on diffraction data alone. Herein, we show that modulated self-assembly can be used to influence the kinetics of self-assembly of Fe-terephthalate MOFs. We comprehensively assess the effect of addition of both coordinating modulators and pH modulators on the outcome of syntheses, as well as probing the influence of the oxidation state of the Fe precursor (oxidation modulation) and the role of the counteranion on the phase(s) formed. In doing so, we shed light on the thermodynamic landscape of this phase system, uncover mechanistics of modulation, provide robust routes to phase pure materials, often as single crystals, and introduce two new Fe-terephthalate MOFs to an already complex system. The results highlight the potential of modulated self-assembly to bring precision control and new structural diversity to systems that have already received significant study.

## Abstract 2

Modulated self-assembly of Fe-terephthalate metal–organic frameworks allows fine control over phase formation, offers insight into kinetic and thermodynamic landscapes, and the discovery of novel materials in an already well-studied complex system.

## Graphical abstract

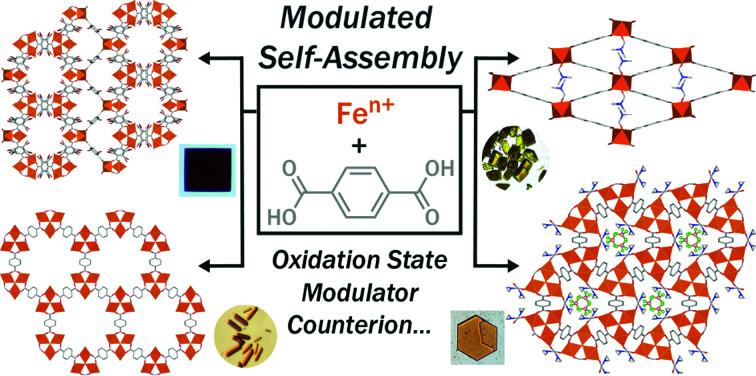

**New concepts**

When synthesising metal–organic frameworks (MOFs), it is usual for multiple phases to result from the same metal–ligand combination. In this study, we focus on the iron terephthalate family of MOFs, and use careful control over synthetic conditions to modulate the self-assembly processes and gain fundamental information on kinetic and thermodynamic landscapes, while discovering even more new members of this well-studied series of MOFs. Our comprehensive approach to modulating self-assembly contrasts with previous work; as well as probing conventional synthetic variables such as reaction time and temperature, we have shown the dramatic effect on phase formation of the addition of modulator molecules, and of the oxidation state and counterion of the Fe precursors. In doing so, we have uncovered robust, reproducible routes to high quality materials for both novel and existing, archetypal MOFs. Our synthetic insights have also highlighted potential pitfalls and offered routes to avoid obtaining and mischaracterising unwanted products from existing literature syntheses. In showing that modulated self-assembly can lead to new materials from well-studied systems, our work demonstrates the structural diversity remaining to be discovered through judicious synthetic control for MOFs and other related network solids such as covalent organic frameworks and even hybrid perovskites.

## 1. Introduction

Metal–organic frameworks (MOFs) – porous networks constructed from inorganic nodes bridged by organic linkers^1^ – are an intensively studied class of materials which show promise in many applications including drug delivery,^2–5^ catalysis,^6–9^ and gas storage.^10–13^ MOFs based on iron^14^ have attracted particular attention for bio-applications due to the endogenous nature of the metal, which makes them desirable as benign carriers for therapeutic drugs.^15,16^ Fe-MOFs are members of a larger subset of porous frameworks linked by trivalent metals^17^ that are typically more robust compared to most MOFs containing divalent metal cations, and thus are desirable for applications where both their low toxicity and relative stability can be exploited.

**Fig. 1:**
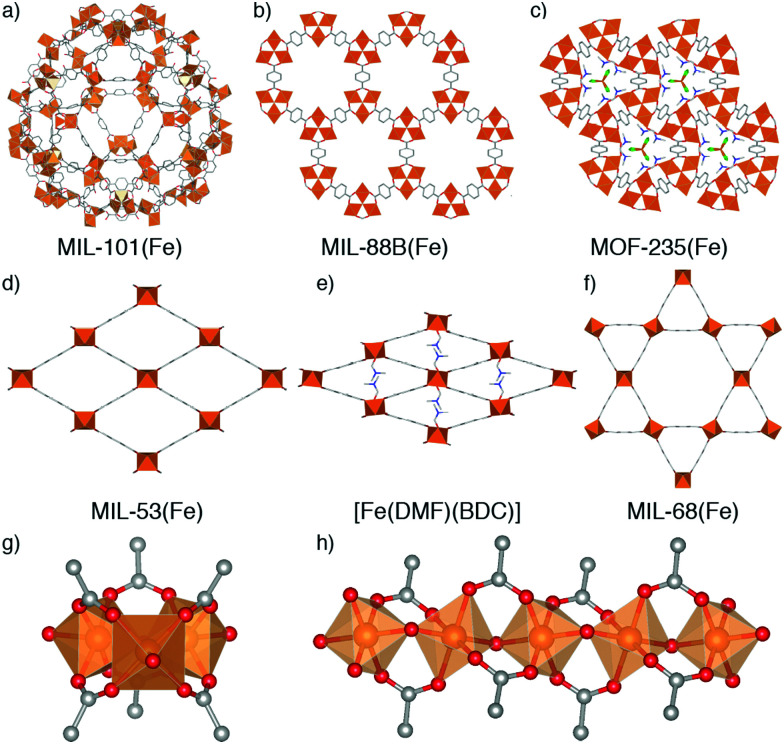
Known Fe-terephthalate phases classified according to inorganic SBU. The top row shows MOFs connected by the trimeric Fe_3_O SBU: (a) MIL-101(Fe), (b) MIL-88B(Fe), and (c) MOF-235(Fe). The middle row shows MOFs connected by one-dimensional chain SBUs: (d) MIL-53(Fe), (e) [Fe(DMF)(BDC)], and (f) MIL-68(Fe). The bottom row shows the two SBUs: (g) [Fe_3_O(RCO_2_)_6_(OH_2_)_2_X], where X is a monoanion such as Cl, F, or OH, and (h) [Fe(μ_2_-OH)(RCO_2_)_2_]_*n*_. Note that in [Fe(DMF)(BDC)], the μ_2_-OH is replaced by an *O*-donor μ_2_-DMF ligand. H atoms removed for clarity.

MOFs where trivalent metals are linked by terephthalate (benzene-1,4-dicarboxylate, BDC) occupy a particularly rich phase space (Fig. 1). There has been significant interest in three well-known Fe^3+^-terephthalate frameworks: MIL-101(Fe), a rigid large pore framework with very high surface area (SA_BET_ up to 4470 m^2^ g^−1^);^18,19^ MIL-88B(Fe), a flexible framework exhibiting continuous breathing upon solvation/desolvation;^20–22^ and MIL-53(Fe), a flexible framework which exhibits well-defined phase transitions between large and narrow pore forms during solvation/desolvation and gas uptake.^23,24^ MIL-101(Fe) and MIL-88B(Fe) are polymorphs with formula [Fe_3_O(BDC)_3_(OH_2_)_2_X], where X is a monoanion typically OH^−^ or Cl^−^, while MIL-53(Fe) has formula [Fe(OH)(BDC)]. In addition, there are another three Fe-terephthalate MOFs which can also crystallise in *N*,*N*-dimethylformamide (DMF) under relatively similar conditions. MIL-68(Fe)^25^ is a large pore polymorph of MIL-53(Fe) with an inflexible Kagomé topology, while [Fe(DMF)(BDC)] is an Fe^2+^ derivative of MIL-53(Fe) where a neutral O-donor DMF ligand replaces the bridging OH of the infinite chain secondary building unit (SBU).^24^

A further example is MOF-235(Fe), with formula [Fe_3_O(BDC)_3_(DMF)_3_][FeCl_4_],^26^ which is topologically identical to MIL-88B(Fe) but contains a pore-located [FeCl_4_]^−^ counterion rather than a cluster bound monoanion, making it particularly challenging to distinguish between these two phases. It is likely due to this complexity, and the similar 2*Θ* values of the main Bragg reflections in powder X-ray diffraction (PXRD) patterns of some of the MOFs, which can also breath (see ESI,† Fig. S1), that we have identified numerous instances where samples of MOF-235(Fe) appear to have been incorrectly assigned both as MIL-88(Fe)^27–30^ and MIL-53(Fe).^31,32^ The implications of performing studies on wrongly assigned phases are quite serious, particularly in cases such as these where the materials possess very divergent properties: MIL-88B(Fe) has a highly flexible structure while MOF-235(Fe) is practically rigid, and MIL-53(Fe) has an entirely different inorganic SBU. It is therefore crucial to establish the effect of tuning particular synthetic parameters on the formation of different members of the Fe-terephthalate phase space, and how such conditions can be manipulated to selectively and reliably synthesise a desired material. This has previously been examined for certain experimental parameters across limited members of the Fe-terephthalate series,^18,33^ but comparison between different studies is hindered by minor variations in synthetic procedures, hence, a comprehensive analysis under controlled conditions is required.

We have previously shown^34^ that control over phase space in Fe-MOFs connected by the extended biphenyl-4,4′-dicarboxylate (BPDC) linker can be exerted by modulated self-assembly.^35^ Using both coordination modulation, the addition of monotopic modulators that mimic the organic ligands, and oxidation modulation, utilising metal starting materials in different oxidation states to those in the product, it is possible to exert kinetic control over self-assembly and select either the non-interpenetrated MIL-88D(Fe) kinetic product or the two-fold interpenetrated MIL-126(Fe) polymorph that is the thermodynamic product.^34^ Coordination modulation has also been used to control the physical properties of Fe-MOFs, such as particle morphology^36–38^ and size.^39,40^ Herein, we apply coordination and oxidation modulation to the synthesis of Fe-terephthalate MOFs, a much more complex system, allowing mapping of the phase space and simple, reproducible isolation of individual phases. In combination with modifying the counterions in the Fe source, we show routes to high quality single crystals of a number of archetypal Fe-terephthalate MOFs and discover a new polymorph of MIL-88B(Fe), suggesting the full structural diversity of these highly-studied materials is yet to be uncovered.

## 2. Results and discussion

### 2.1. Initial modulation scans

**Fig. 2:**
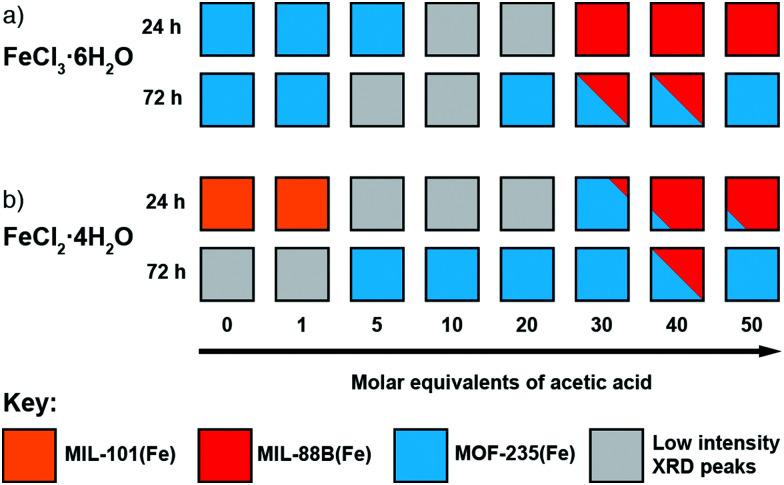
Crystallisation diagrams for syntheses with (a) FeCl_3_·6H_2_O and (b) FeCl_2_·4H_2_O, for both 24 and 72 hours at 120 °C at each modulator concentration. Diffraction data corresponding to the figure can be found in Fig. S2, S3 (part a) and Fig. S4, S5 (part b) (ESI†).

To investigate the phase space, initial reactions were carried out with either FeCl_3_·6H_2_O (1 mmol) or FeCl_2_·4H_2_O (1 mmol), as the differing oxidation state of the Fe precursor previously influenced phase formation with Fe-BPDC MOFs,^34^ and terephthalic acid (1 mmol) in DMF (10 mL) at 120 °C in sealed 50 ml Pyrex reagents jars for either 24 or 72 hours in an isothermal oven (see ESI,† Section S3). DMF plays a complex role in MOF synthesis; thermally decomposing to release a base (dimethylamine) that can deprotonate the linker,^41^ consuming water (a source of O^2−^ and OH^−^ ligands found in SBUs) to produce a potential modulator (formic acid),^42^ and also potentially acting as a structure directing agent.^43^ After allowing to cool naturally to room temperature, the samples were collected by centrifugation and washed with DMF (3 × 20 mL) and then DCM (3 × 20 mL) before drying under vacuum. Subsequently, the samples were analysed using PXRD in order to assess the crystalline phases present. These reactions were carried out either unmodulated, or with the addition of varying amounts of acetic acid as a modulator, to evaluate the effect of coordination modulation on the outcome of synthesis. The results of these experiments are summarised in Fig. 2, which gives a qualitative assessment of the phases present as determined by PXRD (individual diffractograms are given in the ESI,† Fig. S2–S11). The naming system for these samples is FeCl_2_-AA*x*(*T*,*t*) and FeCl_3_-AA*x*(*T*,*t*), where ‘*x*’ equals the number of molar equivalents of acetic acid (AA) added, ‘*T*’ is the synthesis temperature, and ‘*t*’ is the synthesis time.

When FeCl_3_·6H_2_O was used as the metal salt without the addition of modulator, Bragg peaks corresponding to MOF-235(Fe) are present in powder X-ray diffractograms for both 24 and 72 hour syntheses. The addition of acetic acid appears to hinder its formation over 24 hours: at 20 molar equivalents of modulator no corresponding peaks are evident by PXRD, indicating an amorphous phase, and when 30 equivalents or more is used, MIL-88B(Fe) forms. When the synthesis time is extended to 72 hours, MOF-235(Fe) is again the predominant phase, although some MIL-88B(Fe) is present when 30 or 40 equivalents of acetic acid are used; low intensity Bragg reflections are present at intermediate modulator concentrations, suggesting MIL-88B(Fe) is only a minor component that may persist at higher modulator equivalents while not being discernible by diffraction experiments. The presence of MIL-88B(Fe) when higher quantities of acetic acid are present suggests competition with Cl^−^ for Fe cations initially hinders formation of the [FeCl_4_]^−^ counterion necessary to generate MOF-235(Fe). Given that [FeCl_4_]^−^ is stabilised at low pH in aqueous media,^44^ in these DMF-based syntheses acetic acid is seemingly playing a more important role as a ligand (Lewis acid) than a proton donor (Brønsted acid).

For 24 hour syntheses using FeCl_2_·4H_2_O, the unmodulated synthesis displays Bragg peaks which correspond to poor quality MIL-101(Fe); these drop in intensity when 1 equivalent of acetic acid is used and no discernible peaks are evident when 5–20 equivalents of acetic acid are used. With 30 equivalents of acetic acid, MOF-235(Fe) is present alongside a small impurity (a low intensity Bragg peak at 2*θ* = 11°) which cannot be definitively assigned by PXRD, but we postulate to be MIL-88B(Fe) based on the morphology of a minor component observed by scanning electron microscopy (see ESI,† Fig. S9). The samples obtained with 40 and 50 equivalents of acetic acid display peaks corresponding to MIL-88B(Fe) in a manner similar to syntheses with FeCl_3_·6H_2_O as iron source, with small amounts of what is likely to be MOF-235(Fe). The preference for MIL-101(Fe) over MOF-235(Fe) when using FeCl_2_ rather than FeCl_3_ in unmodulated syntheses could again be due to the lower Cl^−^ content impeding formation of the necessary [FeCl_4_]^−^ counterion, although the possibility of the Fe^2+^ source favouring rapid nucleation of a mixed-valence MIL-101(Fe) material should not be ruled out. Formation of MIL-101(Fe) is hindered as modulator concentration increases, suggesting modulation stops the rapid nucleation of this kinetic phase by coordinative competition. For 72 h syntheses with FeCl_2_·4H_2_O, MOF-235(Fe) is again the dominant product regardless of modulator content, but Bragg reflections are weak with 0 or 1 equivalents of modulator.

Regardless of which salt is used, higher acetic acid concentrations favour the formation of MIL-88B(Fe) at 24 hours, but at 72 hours MOF-235(Fe) is the predominant phase under almost all conditions, strongly suggesting that MOF-235(Fe) is the thermodynamic product relative to MIL-88B(Fe). Our previous work with Fe-BPDC MOFs indicated increased modulator content resulted in isolation of the thermodynamic product over the kinetic one, likely by inhibiting rapid nucleation of the kinetic phase through coordinative competition in precursor solutions.^34^ Here, increased acetic acid initially favours MIL-88B(Fe), the kinetic product, likely as acetic acid competes with Cl^−^ for coordination to Fe, disfavouring initial formation of [FeCl_4_]^−^ and thus MOF-235(Fe) at shorter reaction times. As the reaction proceeds, the thermodynamic product, MOF-235(Fe) is the result. For both FeCl_2_·4H_2_O and FeCl_3_·6H_2_O, the crystallinity of the products generally increases as the concentration of acetic acid is increased, demonstrating the effective role of acetic acid as a modulator in these systems.

### 2.2. Assessing the kinetic and thermodynamic relationships between phases

**Fig. 3:**
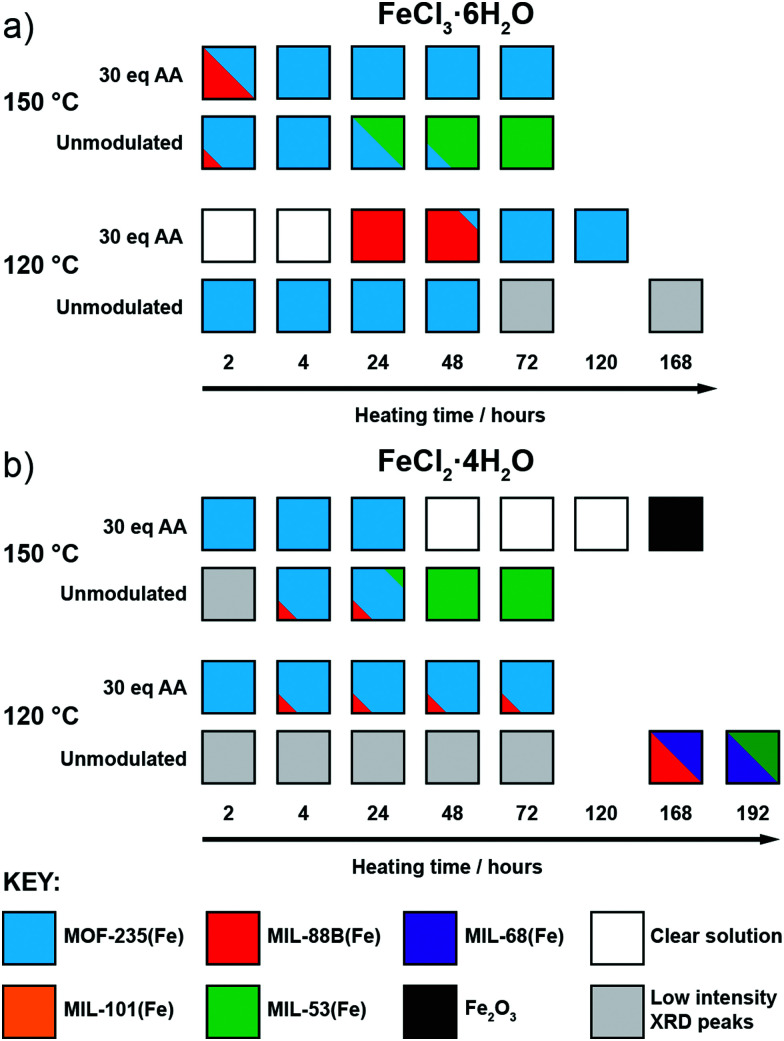
Crystallisation diagrams for syntheses with (a) FeCl_3_·6H_2_O and (b) FeCl_2_·4H_2_O across different times and temperatures, with and without 30 eq. acetic acid modulator. Diffraction data corresponding to the figure can be found in Fig. S12–S15 (part a) and S16–S22 (part b) (ESI†).

After observing the formation of these phases controlled by modulator concentration at two reaction times, we focused on exploring this over more time points. Thus, additional reactions were carried out with reaction times fixed between 2 hours and 3 days (in some cases, even longer reaction times were used), both with and without the addition of 30 eq. of acetic acid, as this intermediate modulator concentration consistently yielded either MOF-235(Fe) or MIL-88B(Fe) during 24 and 72 hour reactions. These reactions were also carried out at both 120 °C and 150 °C to probe the effect of temperature in this system, with the qualitative results in Fig. 3 based on interpretation of individual diffractograms in the ESI,† Fig. S12–S27.

When using FeCl_3_·6H_2_O (Fig. 3a) and no modulator at 120 °C, MOF-235(Fe) begins to form after 2 hours, alongside a minor amount of MIL-88B(Fe), and peaks in crystallinity after 4 hours. After 24 hours the crystallinity seemingly drops, as Bragg peak intensities weaken and continue to drop with extended heating, which may be due to a transformation to smaller crystallites with only short-range ordering, but MOF-235(Fe) remains the only product formed. In contrast, the addition of acetic acid prevents any solid formation after 4 hours at 120 °C, and then leads to the formation of MIL-88B(Fe) after 24 hours, which transitions to highly crystalline MOF-235(Fe) after 72 hours and onwards. This could be either the modulator controlling the kinetics of self-assembly through coordinative competition, or acetic acid inhibiting formation of [FeCl_4_]^−^ (or indeed a combination of both) as described previously.

Analogous acetic acid modulated syntheses using FeCl_2_·4H_2_O (Fig. 3b) also generate highly crystalline MOF-235(Fe) for reactions up to 72 hours, but a persistent additional phase can be seen by PXRD after 4 hours, which we expect corresponds again to MIL-88B(Fe). The presence of small amounts of persistent MIL-88B(Fe) could be a consequence of the lower Cl^−^ content of the FeCl_2_·4H_2_O starting material inhibiting the formation of the FeCl_4_^−^ counterion of MOF-235(Fe).

For both salts at 150 °C without a modulator, MOF-235(Fe) is predominantly formed at shorter reaction times alongside a minor MIL-88B(Fe) component, although it takes slightly longer to form with FeCl_2_·4H_2_O than with FeCl_3_·6H_2_O, likely due to the lower Cl^−^ content. Large yellow rod-shaped crystals typically appear after 48 hours; after a 72 hour synthesis with FeCl_2_·4H_2_O, a suitable crystal was characterised by single-crystal X-ray diffraction (SCXRD) and found to possess the already well-known MIL-53(Fe) structure consisting of infinite chains of Fe(OH) linked together by terephthalates to give diamond-shaped channels (see ESI,† Section S4.2). In this crystal structure, the channels run down the crystallographic *a* axis and are occupied by disordered DMF molecules which H-bond to the bridging OH ligand; a previously reported crystal structure had pyridine as guest in a similar manner.^24^ PXRD revealed that after subsequent solvent exchange and drying from dichloromethane (DCM), the hydrated phase of MIL-53(Fe), known as MIL-53(Fe)_lt^23^ is obtained (see ESI,† Fig. S25), and this is the sole phase present after 72 hours reaction and work up. Despite using an Fe^2+^ precursor, no evidence is seen for the analogous Fe^2+^ phase, [Fe(DMF)(BDC)]_*n*_, only the Fe^3+^-linked MIL-53(Fe). Since only crystals of MIL-53(Fe) are present after 72 hours, and any MOF-235(Fe) which appears to form before then is absent, it can be assumed that the phase transformation from MOF-235(Fe) to MIL-53(Fe) is a dissolution and recrystallization process – this has been previously proposed based on time-resolved energy-dispersive X-ray diffraction studies of their crystallisation.^45^ It is also suggestive that MIL-53(Fe), the denser of the two phases, is the thermodynamic product relative to MOF-235(Fe).

When acetic acid is added to syntheses, there is distinctly different behaviour between the two salts at 150 °C. For reactions with FeCl_3_·6H_2_O, a small amount of MIL-88B(Fe) forms within 2 hours but is absent after 4 hours, after which only highly crystalline MOF-235(Fe) is evident by PXRD. For syntheses with FeCl_2_·4H_2_O, MOF-235(Fe) again forms rapidly, but unlike FeCl_3_·6H_2_O syntheses, a complete dissolution is evident after 48 hours. On extended reaction times (168 hours), solid Fe_2_O_3_ is obtained, as confirmed by PXRD, rather than a MOF product. One possible explanation might be that with a lower concentration of chloride ions in solution, the long-term stability of MOF-235(Fe) in the synthesis mixture is lower when using FeCl_2_·4H_2_O, and thus it eventually breaks down.

Finally, at 120 °C and without a modulator present, reactions with FeCl_2_·4H_2_O did not yield a highly crystalline product until 168 hours, at which point MIL-68(Fe) forms, as evidenced by PXRD. MIL-68(Al) is known to be a kinetically favoured intermediate polymorph relative to MIL-53(Al),^46^ and we expect this relationship to be the same for the Fe analogues. The sample contained large needle-like crystals which presumably correspond to this phase, as well as some orange powder. The PXRD pattern shows that this sample contains a phase impurity with Bragg peaks similar to those seen for MIL-88B(Fe), which is consistent with the hexagonal needle morphology of the orange powder observed by optical microscopy. An attempt to isolate the crystals in phase-pure form was conducted by slightly increasing the reaction time (192 hours) and recovering the crystals by removing the suspension and replacing with fresh DMF. PXRD analysis of this sample (see ESI,† Fig. S22) shows that there is a mixture of MIL-68(Fe) and MIL-53(Fe). As both samples contain the same Fe(OH) infinite chain SBU, it is likely that MIL-68(Fe) converts over time to the denser MIL-53(Fe) structure, confirming MIL-68(Fe) is the kinetic phase of the two. These two syntheses provide context for the thermodynamic relationship between MIL-68(Fe) and other phases, giving an order, excluding MOF-235(Fe), of MIL-101(Fe) < MIL-88B(Fe) < MIL-68(Fe) < MIL-53(Fe).

Across all experiments, no formation of MIL-53(Fe) is evident when acetic acid is present in syntheses, which indicates that coordination modulation favours formation of discrete [Fe_3_O(RCO_2_)_6_] SBUs (Fig. 1g) over infinite 1D chain SBUs (Fig. 1h), perhaps through templation, reminiscent of the preformed SBU approach to MOF synthesis.^39,47^

### 2.3. Summary

The results show that MOF-235(Fe) will eventually convert to MIL-53(Fe) given a sufficient amount of reaction time 150 °C, likely in a dissolution/recrystallization process, and thus MIL-53(Fe) can indeed be assumed to be the thermodynamically favoured product relative to MOF-235(Fe), giving a stability order, excluding MIL-68(Fe), of MIL-101(Fe) < MIL-88B(Fe) < MOF-235(Fe) < MIL-53(Fe). This is supported by the fact that MIL-53(Fe) is observed only with long synthesis times (>1 week) at 120 °C or relatively short times (2 days) at 150 °C. The presence of acetic acid clearly impedes the formation of MIL-53(Fe), likely by favouring the formation and stabilisation of the discrete [Fe_3_O(RCO_2_)_6_] cluster. Subsequent reactions using HCl as a pH modulator instead of acetic acid as a coordination modulator give MIL-53(Fe) as the sole product, regardless of the salt used or the temperature (see ESI,† Fig. S27). This is consistent with previous studies on the synthesis of the iron amino-terephthalate analogues^18^ and also lends more credence to the hypothesis that acetic acid plays a greater phase-directing role through SBU templation, *i.e.* acting as a coordination modulator, than merely modulating the pH and influencing the kinetics of crystallisation by inhibiting linker deprotonation.

MOF-235(Fe) forms under nearly all conditions save for FeCl_2_-AA0(120 °C,*t*) and is often the final product formed at lower temperatures or in the presence of acetic acid modulator, again suggesting that it is the thermodynamically preferred product over MIL-88B(Fe). When considering that [FeCl_4_]^−^ is required to form MOF-235(Fe), it is unsurprising that it forms more readily when using FeCl_3_·6H_2_O than FeCl_2_·4H_2_O without a modulator, as this increases the Cl^−^ concentration, however reactions with FeCl_2_·4H_2_O seem to reach MOF-235(Fe) faster than analogous syntheses with FeCl_3_·6H_2_O syntheses in the presence of acetic acid, suggesting the oxidation state of the metal does play a significant role in the kinetics of self-assembly.

These results contradict the conclusions of a previous study that investigated the formation of MOF-235(Fe) *vs.* MIL-88B(Fe) using single metal and mixed-metal approaches.^48^ In this study, it was shown that MIL-88B(Fe) forms after MOF-235(Fe) in single metal syntheses, however, their synthesis includes the use of NaOH (0.8 eq.) which has already been reported to favour the formation of MIL-88B(Fe).^18^ Since it is unclear exactly what effect NaOH has on the synthesis – it will favour deprotonation of the terephthalic acid but OH^−^ may also compete with Cl^−^ for coordination to Fe and hinder formation of [FeCl_4_]^−^ – our results across different timescales give a clearer indication of the thermodynamic preference.

### 2.4. Variation of the Fe-precursor

It is clear that the role of counterion is key in the formation of phases such as MOF-235(Fe). As such, the next step was the use of alternative Fe sources. While chlorides are typically the most commonly used in the literature, there are a plethora of other common and inexpensive iron salts which can also be used to synthesise Fe-MOFs, and so iron(iii) nitrate nonahydrate, iron(ii) tetrafluoroborate hexahydrate, and iron(ii) acetate were all employed in similar syntheses to those using the iron chlorides (see ESI,† Section S5). Initial acetic acid modulation scans were conducted at 120 °C for 24 hours, and additional reactions were carried out at 150 °C for 72 hours without a modulator, as these conditions had been found to be sufficient to reach the thermodynamic product, MIL-53(Fe), in the previous experiments. The naming system used is Fe(counterion)-AA*x*(*T*,*t*) where ‘*x*’ equals the number of molar equivalents of acetic acid (AA) added, ‘*T*’ is the synthesis temperature, and ‘*t*’ is the synthesis time.

For syntheses using Fe(NO_3_)_3_·9H_2_O (see ESI,† Fig. S28–S30), at a synthesis temperature of 120 °C and reaction time of 24 hours, crystalline materials could only be obtained with 20 eq. or more of acetic acid; these correspond to MIL-88B(Fe). Increasing the reaction times to 72 hours did not yield a significant improvement for most of the modulator concentrations, and for 20 eq. of acetic acid the crystallinity drops significantly. The 150 °C synthesis without modulator yielded only amorphous material, similar to those at 120 °C, whereas analogous syntheses with Fe chlorides yielded MIL-53(Fe), which further suggests that chloride aids formation of this phase. Under these conditions, Fe(NO_3_)_3_·9H_2_O offers less structural diversity in Fe-terephthalate MOFs.

**Fig. 4:**
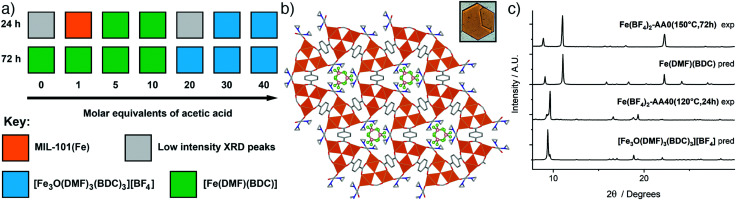
(a) Crystallisation diagrams for syntheses with Fe(BF_4_)_2_·6H_2_O at 120 °C at 24 h and 72 h. Diffraction data corresponding to the figure can be found in Fig. S31 and S32 (ESI†). (b) Packing structure of [Fe_3_O(DMF)_3_(BDC)_3_][BF_4_] viewed down the *c* axis, with an image of a hexagonal plate crystal inset. C: gray; O: red; N: blue; B: pink; F: green; Fe: orange spheres; H atoms removed for clarity. (c) Stacked powder X-ray diffractograms for samples of both phases compared to those predicted for [Fe_3_O(DMF)_3_(BDC)_3_][BF_4_] and [Fe(DMF)(BDC)]. Some preferred orientation is evident in diffractograms of [Fe_3_O(DMF)_3_(BDC)_3_][BF_4_] due to the flat plate crystal morphology.

Synthesis with Fe(BF_4_)_2_·6H_2_O at 120 °C primarily yielded two distinct phases as seen in the reaction summary in Fig. 4a (diffraction data are provided in the ESI,† Fig. S31 and S32). With the incorporation of up to 10 equivalents of acetic acid, [Fe(DMF)(BDC)] is the product, with longer reaction times favouring its formation. Analogous synthesis at 150 °C without a modulator yielded large yellow crystals, suitable for single crystal X-ray diffraction, after 3 days. The structure is identical to the already-reported [Fe(DMF)(BDC)] structure (YAXBUV in the CCDC)^24^ which is similar to MIL-53(Fe) but in this case the bridging hydroxides are replaced by DMF molecules and the Fe ions are in the 2+ oxidation state. Interestingly, we found that the PXRD pattern of bulk sample Fe(BF_4_)_2_-AA0(150 °C,72 h) changes upon drying from DCM (see ESI,† Fig. S33), suggesting it retains the flexibility of its Fe^3+^ analogue MIL-53(Fe). We have not yet, however, been able to extract structural information, and the presence of coordinated DMF in the MOF means the possibility of sample degradation cannot be ruled out.

In contrast, incorporation of higher amounts of acetic acid to syntheses yields a new phase. Synthesis with Fe(BF_4_)_2_·6H_2_O at 120 °C for 24 h using 40 eq. of acetic acid modulator yielded large hexagonal plate crystals, which were suitable for SCXRD. The structure consists of [Fe_3_O(DMF)_3_(RCO_2_)_6_] SBUs bridged by terephthalates into a MIL-88 topology (**acs**) MOF with hexagonal channels that run down the crystallographic *c* axis (Fig. 4b). These channels are occupied by disordered [BF_4_]^−^ anions that are present in a 1 : 3 ratio relative to Fe, giving an overall framework formula of [Fe_3_O(DMF)_3_(BDC)_3_][BF_4_]; hence the structure is very similar to MOF-235(Fe), except with a [BF_4_]^−^ anion in place of [FeCl_4_]^−^. The carbonyl carbon in the coordinated DMF molecule is disordered between two positions, the BF_4_^−^ anion is positionally and rotationally disordered, more so than [FeCl_4_]^−^ in MOF-235(Fe), likely as it is a smaller anion and thus a poorer fit for the pore cavity. Bond valence sum calculations give a value of 3.070 for the Fe atoms of the SBU, confirming that it is autoxidised during the reaction to the 3+ oxidation state. Comparison between the predicted and experimental PXRD patterns confirm that the bulk of the Fe(BF_4_)_2_-AA40(120 °C,24 h) sample corresponds to [Fe_3_O(DMF)_3_(BDC)_3_][BF_4_] (Fig. 4c).

From a formula perspective, both contain coordinated DMF molecules but differ primarily in their oxidation states – [Fe(DMF)(BDC)] contains Fe^2+^ and [Fe_3_O(DMF)_3_(BDC)_3_][BF_4_] contains Fe^3+^ – and the presence of [BF_4_]^−^. The kinetic and thermodynamic relationship between these two structures is therefore harder to establish from these experiments, as there is no case where both phases are crystallised from an identical synthesis mixture at different temperatures or over different synthesis times; it is the modulator which controls phase (Fig. 4a). The addition of acetic acid clearly favours [Fe_3_O(DMF)_3_(BDC)_3_][BF_4_], which contains a discrete [Fe_3_O] SBU, over [Fe(DMF)(BDC)] with its infinite one-dimensional chain SBU. This mirrors the relationship previously described between the analogous phases MOF-235(Fe) (kinetic) and MIL-53(Fe) (thermodynamic), but could be a cluster templating effect rather than acetic acid modulating the kinetics, particularly as (i) [Fe(DMF)(BDC)] is obtained at both 120 °C and 150 °C in the absence of acetic acid, and (ii) formation of [Fe_3_O(DMF)_3_(BDC)_3_][BF_4_] requires oxidation of the Fe^2+^ starting material, another kinetic barrier. Modulated self-assembly, however, does make it possible to isolate bulk, phase pure samples of either material.

When using Fe(OAc)_2_ as starting material, two phases can be observed by PXRD when using a temperature of 120 °C and a synthesis time of 24 hours (see ESI,† Fig. S34). Bragg peaks corresponding to the dried sample of [Fe(DMF)(BDC)] are seen with 0–10 eq. of AA, and then MIL-88B(Fe) is present as a highly-crystalline phase from 20–50 eq. Fe(OAc)_2_ being an Fe^2+^ salt likely favours the formation of [Fe(DMF)(BDC)] at low modulator concentrations, similar to what was seen with Fe(BF_4_)_2_·6H_2_O, whereas at higher concentrations the equilibrium shifts towards favouring the Fe_3_O(RCO_2_)_6_ clusters seen in MIL-88B(Fe), as we have rationalised for modulation scans with the iron chloride salts.

The choice of metal precursor therefore has a profound effect on which phase crystallises; MIL-88B(Fe) is the product from reactions with Fe(NO_3_)_3_, FeCl_3_, or Fe(OAc)_2_ and terephthalic acid after heating for 24 hours at 120 °C when a sufficient quantity of acetic acid (>30 eq.) is added to the synthesis. Fe(BF_4_)_2_ is the only Fe-source that does not ever yield MIL-88B(Fe), regardless of modulator concentration or time, which suggests that [Fe_3_O(DMF)_3_(BDC)_3_][BF_4_] and MOF-235(Fe) are formed preferentially over MIL-88B(Fe) when there is a suitable anion to enable their formation. For the Fe^2+^ salts Fe(OAc)_2_ and Fe(BF_4_)_2_, it seems that the use of a carboxylate modulator favours the formation of the Fe_3_O cluster (Fe^3+^), while at lower modulator concentrations the [Fe(DMF)(BDC)] phase is predominant. Finally, MIL-53(Fe) syntheses seem to require the presence of Cl^−^, as well as high temperatures to avoid MOF-235(Fe) formation.

### 2.5. FeSO_4_·7H_2_O as the Fe-precursor

**Fig. 5:**
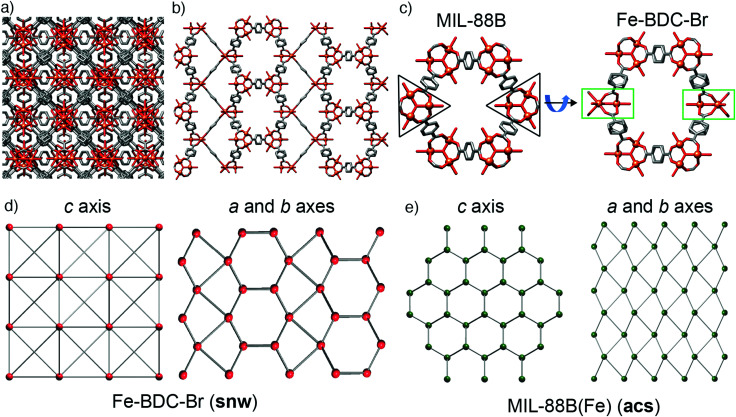
(a) Crystal packing in the solid-state structure of Fe–BDC-Br as viewed down (a) the *c* axis, and (b) the equivalent *a* and *b* axes. (c) The relationship between the hexagonal units with respect to SBU orientation in the crystal structure of Fe–BDC-Br compared to MIL-88B. Disordered Br atoms in Fe–BDC-Br and H atoms in both removed for clarity. Topological representations of (d) Fe–BDC-Br (**snw**) compared to (e) MIL-88(Fe) (**acs**).

During this study, we also explored FeSO_4_·7H_2_O as starting material, being both a source of Fe^2+^ and having a counteranion that is tetrahedral, like BF_4_^−^ and FeCl_4_^−^, but is also a dianion. Acetic acid modulation did not, however, generate an analogue of MOF-235(Fe) with an alternative counterion (SO_4_^2−^ or HSO_4_^−^), but large, high quality single crystals of MIL-88B(Fe) (see ESI,† Section S5.4), which has previously required the use of HF to grow sufficiently large crystals for SCXRD.^49^ The structure is identical to the reported single crystal structure,^49^ crystallising in the *P*63/*mmc* space group with *a* = 13.911(1) Å and *c* = 17.661(1) Å, and will also crystallise from unmodulated syntheses. This simple protocol to isolate single crystals of MIL-88(Fe) led us to explore substituted terephthalates, hoping to crystallographically characterise an isoreticular series, and under these conditions we found we could also grow large crystals using 2-bromo-1,4-benzenedicarboxylate (BDC-Br) as the linker (see ESI,† Section S5.5). These had a clearly different crystal habit to MIL-88B(Fe), being rectangular as opposed to hexagonal, reflecting that the framework, which will be referred to as Fe–BDC-Br, crystallises in the tetragonal space group *I*4_1_/*amd* with *a* = 16.307(1) Å and *c* = 52.852(4) Å. The tetragonal arrangement of overlaid clusters in the packing structure of Fe–BDC-Br is clearly visualised (Fig. 5a) down the crystallographic *c* axis (4-fold). Structurally, both Fe–BDC-Br and MIL-88B(Fe) share similar building blocks with the same connectivity: [Fe_3_O(RCO_2_)_6_] SBUs connected by terephthalates. Fe–BDC-Br possesses hexagonal channels very similar to those in MIL-88B(Fe) which run down the equivalent *a* and *b* axes alternatively (Fig. 5b), while in MIL-88B(Fe) there are hexagonal channels running down only the *c* axis (6-fold). Considering both frameworks as viewed down a single hexagonal channel (Fig. 5c), in MIL-88B(Fe) all of the trigonal SBUs face in the same direction, while in Fe–BDC-Br two of the SBUs are rotated by 90° such that they sit perpendicular to the rest. This disrupts the hexagonal symmetry of MIL-88B(Fe), and so looking down the *a* or *b* axes of Fe–BDC-Br (Fig. 5b) it is possible to see bands of both the structural elements corresponding to the hexagonal (*c* axis) and the linear (*a* and *b* axes) of MIL-88B(Fe), almost reminiscent of twinning at an ordered, atomic level.

The structural similarities are even more apparent in the topological analyses of the frameworks, which were carried out using the ToposPro program.^50^ The newly synthesised Fe–BDC-Br framework displays the **snw** underlying unimodal net topology with a vertex symbol of 4·4·4·4·4·4·4_2_·4_2_·4_2_·6_4_·6_4_·6_4_·6_4_·6_4_·6_4_. Comparing the **snw** topology (Fig. 5d) to the underlying **acs** net of MIL-88B(Fe), (Fig. 5e) the two are identical in connectivity, although differ in coordination sequence, which distinguishes them. The most symmetric embedding of **snw** is the *I*4_1_/*amd* tetragonal space group, which is lower in symmetry than hexagonal *P*6_3_/*mmc* space group of MIL-88B(Fe), suggesting that **snw** is a subnet of the **acs** net. One notable difference between the two MOFs is that in Fe–BDC-Br, bond valence calculations (see ESI,† Section S5.6) suggest the cluster is mixed valence [Fe^III^_2_Fe^II^O(RCO_2_)_6_], while for MIL-88B(Fe) the cluster is single valence [Fe^III^_3_O(RCO_2_)_6_], however, this change in valence does not account for the topological differences between the two MOFs.

There is also a strong dependence between the Fe-source used in the synthesis and the product which forms when using the 2-bromoterephthalate linker. When using both ferrous and ferric chloride, MIL-88B(Fe)-Br (**acs**) is the sole product, while both FeSO_4_·7H_2_O and Fe(OAc)_2_ yield the novel Fe–BDC-Br (**snw**) phase, regardless of the synthesis conditions (time, temperature, addition of acetic acid). As such, unlike the terephthalate phase space, it is unclear to us which of the two phases is thermodynamically more stable or why there is such a strong dependence between the Fe-source and the phase which forms: the Fe–BDC-Br crystal structure does not contain or appear to require the sulfate or acetate anions for its formation. However, its mixed valence cluster does not require a monoanion (OH^−^ or Cl^−^) for charge balance, which we assume is required for formation of MIL-88B(Fe)-Br, and may explain the formation of the latter with iron chloride salts. We have not, however, been able to isolate this phase with unsubstituted terephthalate.

To understand why MIL-88B(Fe) (**acs**) is seemingly formed over the new Fe–BDC (**snw**) phase when the cluster is of single Fe^3+^ valence, we performed hybrid density functional theory (DFT) calculations (see ESI,† Section S6) of both MIL-88B(Fe) (**acs**) and Fe–BDC (**snw**) using the unfunctionalised BDC linker, to remove issues regarding disorder of the bromine group in the 2-bromoterephthalate-based MOFs. Our hybrid DFT calculations indicate that MIL-88B(Fe) (**acs**) is energetically more stable than Fe–BDC (**snw**) by 9.8 kJ mol^−1^ per Fe_3_O cluster, which means MIL-88B(Fe) (**acs**) could indeed be a thermodynamic product over Fe–BDC (**snw**) when the cluster is of single Fe^3+^ valence and the linker is unsubstituted terephthalate. We also performed hybrid DFT calculations on the MOFs featuring mixed valence Fe^2+^/Fe^3+^ clusters, in both **acs** and **snw** topologies, and our calculations indicate that the energy difference between MIL-88B(Fe) (**acs**) and Fe–BDC (**snw**) is reduced to only 2.3 kJ mol^−1^ per Fe_3_O cluster, suggesting the new Fe–BDC (**snw**) phase is likely to be stabilised by the complex electronic structure featuring mixed Fe^2+^ and Fe^3+^ ions. In addition, we suspect a range of other factors which were not accounted for in our hybrid DFT calculations, including Br substitution on the linker, solvent effects and vibrational entropy, may have also contributed to the experimental formation of Fe–BDC-Br **(snw)** rather than MIL-88B(Fe)-Br (**acs**).

It is both surprising and intriguing to have discovered a new structure in a phase space which has already been explored so extensively, but also that it possesses a rarely seen topology. This highlights how much phase complexity is perhaps missed, or even omitted, during many conventional synthetic studies, and we expect that with the arrival and implementation of automation^51^ and machine learning^52^ in combination with new modulated self-assembly protocols,^35^ that this will become even more evident for other MOF systems.

## 3. Conclusions

**Fig. 6:**
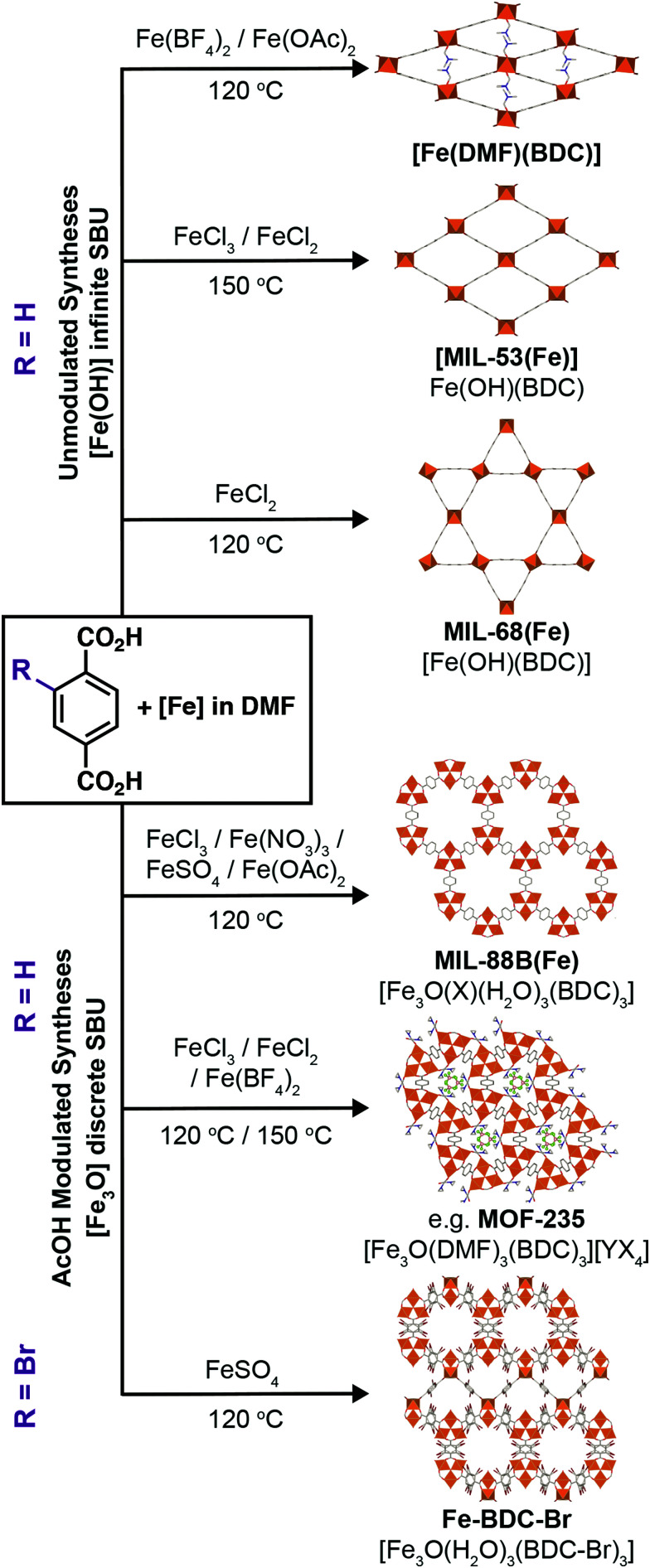
Scheme showing the main phases which can be obtained from each metal precursor, both unmodulated and with the addition of acetic acid.

We have explored the phase space of Fe-terephthalate MOFs and found reliable, reproducible routes to various MOFs containing the chain and trigonal SBUs, many as single crystal samples, as well as gaining some insight into the kinetic/thermodynamic relationships between them. Our experiments have allowed us to confirm the relative stabilities of MIL-68(Fe) and MOF-235(Fe) with respect to the other Fe^3+^-terephthalate phases that can form, with the kinetic/thermodynamic relationship between the two the only unresolved question in this series. Compared to our previous work with Fe–BPDC, the effect of varying the oxidation state of the metal precursor is much more complex, as the counterion dictates the nature of the phases which can form as well as preferentially favouring one over another. We have demonstrated that MOFs with the discrete [Fe_3_O(RCO_2_)_6_] trigonal SBU can be best stabilised by the addition of a monocarboxylate modulator, acetic acid, while MOFs with the chain SBU can best be obtained without modulator or by use of a mineral acid such as HCl (Fig. 6). Time is also a crucial factor in these syntheses, as for a given reaction mixture, some phases are transient and can dissolve or even lose their crystallinity over time.

By modifying the counteranion in the Fe precursor, we have also isolated two new Fe-terephthalate phases, [Fe_3_O(DMF)_3_(BDC)_3_][BF_4_] and the novel polymorph of MIL-88B(Fe), termed Fe–BDC-Br. The formation of the former can be rationalised by the anion incorporation, whilst the latter seems to rely on the use of an Fe^2+^ salt to form a mixed valence cluster and a non-coordinating anion that will not template a MOF analogous to the former. Modulation can again play a role here, suggesting that hidden structural diversity is waiting to be discovered in other well-studied archetypal MOF families, and that undiscovered polymorphism in MOFs could yield materials with novel, desirable properties.^53^

## Conflicts of interest

The authors declare no competing financial interest.

## Supplementary Material

MH-008-D1MH01663F-s001

MH-008-D1MH01663F-s002
