# Supplementary material for: Exploring and expanding the Fe-terephthalate metal–organic framework phase space by coordination and oxidation modulation
Source: Mater Horiz. 2021 Oct 13;8(12):3377–86. doi: 10.1039/d1mh01663f (PMC8628537; doi:10.1039/d1mh01663f)
Supplement: MH-008-D1MH01663F-s001 [file MH-008-D1MH01663F-s001.pdf]

Electronic Supplementary Material (ESI) for Materials Horizons.
This journal is © The Royal Society of Chemistry 2021

Exploring and Expanding the Fe-Terephthalate Metal-Organic Framework Phase Space by Coordination and Oxidation Modulation

Dominic Bara,[1] Emily G. Meekel,[1] Ignas Pakamorė,[1] Claire Wilson,[1] Sanliang Ling,[2] Ross S. Forgan[1]*

1. WestCHEM School of Chemistry, University of Glasgow, Joseph Black Building, University Avenue, Glasgow G12 8QQ, UK.

2. Advanced Materials Research Group, Faculty of Engineering, University of Nottingham, University Park, Nottingham NG7 2RD, UK.

Email: Ross.Forgan@glasgow.ac.uk
Web: www.forganlab.com

SUPPORTING INFORMATION

Table of Contents

S.1. GENERAL EXPERIMENTAL METHODS **S3**

S.2. ALL PREDICTED PXRD PATTERNS **S4**

S.3. SYNTHESIS WITH VARIABLE MODULATOR CONCENTRATION **S5**

S3.1. GENERAL CONDITIONS FOR SYNTHESIS **S5**

S3.2. PXRD DATA **S6**

S.4. SYNTHESIS WITH VARIABLE HEATING TIME **S15**

S4.1. GENERAL CONDITIONS FOR SYNTHESIS **S15**

S4.2. SINGLE-CRYSTAL SYNTHESIS OF MIL-53(FE)-DMF **S25**

S4.3. PXRD DATA **S27**

S.5. SYNTHESIS WITH VARYING FE-PRECURSOR **S30**

S5.1. GENERAL CONDITIONS FOR SYNTHESIS **S30**

S5.2. SINGLE-CRYSTAL SYNTHESIS OF [FE(DMF)(BDC)] **S34**

S5.3. SINGLE-CRYSTAL SYNTHESIS OF [$FE_3O(DMF)_3(BDC)_3$][BF_4] **S35**

S5.4. SINGLE-CRYSTAL SYNTHESIS OF MIL-88B(FE) **S37**

S5.5. SINGLE-CRYSTAL SYNTHESIS OF FE-BDC-BR **S37**

S5.6. VALENCE BOND SUM (BVS) CALCULATIONS FOR MIL-88B(FE) AND FE-BDC-BR **S38**

S.6. DFT CALCULATIONS **S40**

S.7. REFERENCES **S41**

S.1. General Experimental Methods

All starting materials were purchased from commercial sources and used without further purification.

Powder X-Ray Diffraction (PXRD): PXRD measurements were carried out at 298 K using primarily a PANalytical X'Pert PRO diffractometer (λ (CuKα) = 1.5405 Å) on a mounted bracket sample stage, and the pulse height discrimination (PHD) lower level was raised to 55% to filter out some of the low-energy X-rays caused by fluorescence from iron. All other measurements were carried out using a Rigaku MiniFlex diffractometer (λ (CuKα1) = 1.54056 Å, (CuKα2) = 1.54439 Å) on a spinning zero-background holder. All indexing and Pawley fitting was carried out using GSAS-II.[S1]

Single Crystal X-Ray Diffraction (SCXRD): Data were collected using a Bruker D8 VENTURE diffractometer equipped with Photon II CPAD detector, dual IμS 3.0 Cu and Mo sources and an Oxford Cryosystems N-Helix device.

Scanning Electron Microscopy (SEM): Powders were deposited onto carbon tabs mounted on aluminium stubs, these were subsequently coated with Pd for 150 seconds using a Polaron SC7640 sputter coater. Samples were imaged using a Carl Zeiss Sigma variable Pressure Analytical SEM with Oxford Microanalysis.

Thermogravimetric Analysis (TGA): Measurements were carried out using a TA Instruments Q500 Thermogravimetric Analyser. Measurements were collected from room temperature to 600 °C with a heating rate of 10 °C min^{-1} under an air atmosphere.

S.2. All Predicted PXRD Patterns

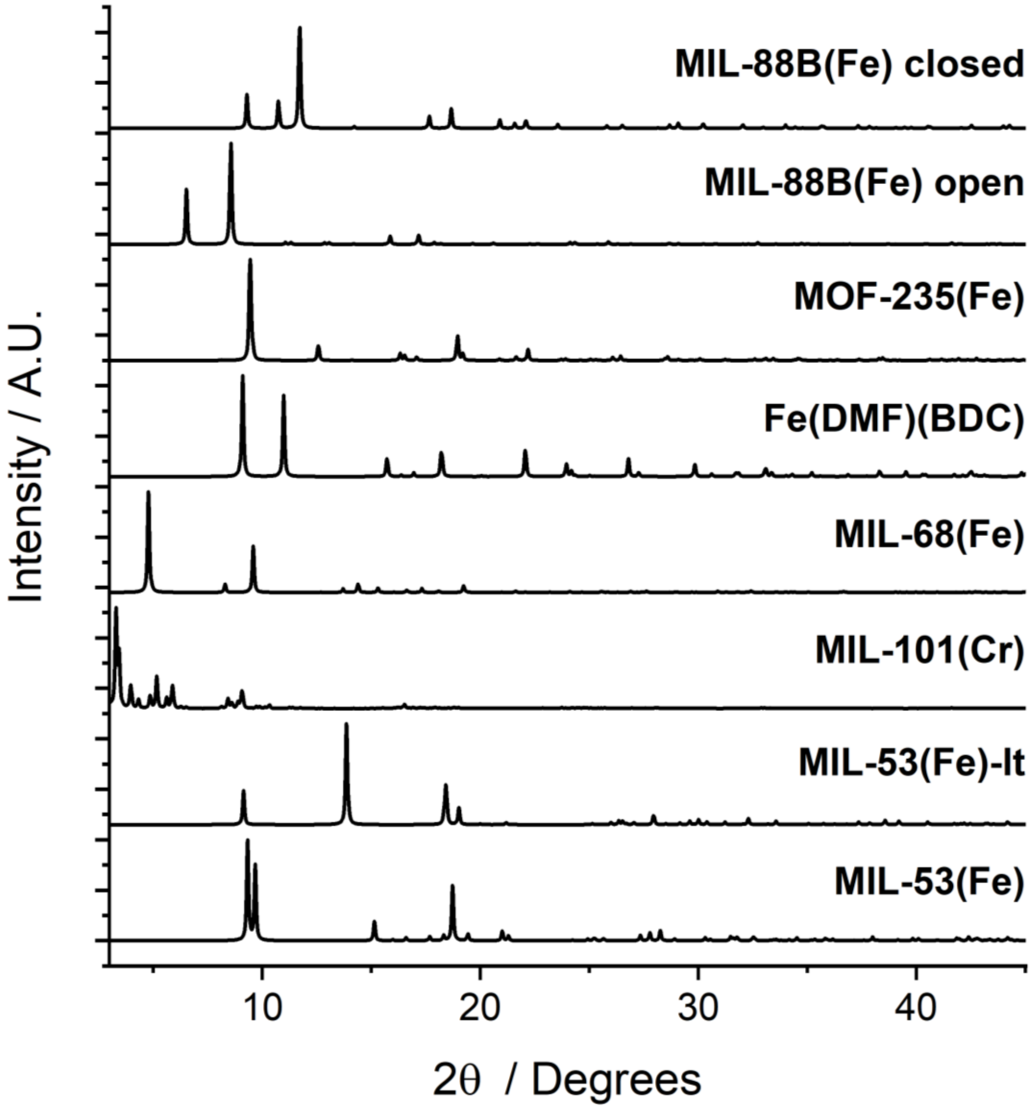

Figure S1. Predicted PXRD patterns of MIL-53(Fe),[S2] MIL-53(Fe)-lt,[S3] MIL-101(Cr),[S4] MIL-68(Fe),[S5] Fe(DMF)(BDC),[S2] and MOF-235(Fe).[S6] and MIL-88B(Fe) (open and closed pore forms).[S7] The predicted PXRD pattern for MIL-101(Cr) is used, as there is no freely available data for MIL-101(Fe), and the two MOFs are isostructural, particularly as MIL-101 is not flexible.

S.3. Synthesis with Variable Modulator Concentration

S3.1. General Conditions for Synthesis

Either iron(II) chloride tetrahydrate or iron(III) chloride hexahydrate (1 mmol) and terephthalic acid (1 mmol) were added to a 50 mL Pyrex reagent jar and DMF (10 mL) was added, for modulated samples acetic acid (1-50 mmols) was also added. The jar was capped and sonicated until the solids dissolved before heating in an isothermal oven at 120 °C for either 24 or 72 hours. After allowing to cool to room temperature, the suspension was separated by centrifuging. The supernatant was decanted and fresh DMF (20 mL) was added before centrifuging again. This was repeated three times, before repeating the procedure another three times with DCM (20 mL). The sample was then dried overnight in a desiccator under vacuum. The naming system for these samples is **$FeCl_2$-AAx(T,t)** and **$FeCl_3$-AAx(T,t)**, where 'x' equals the number of molar equivalents of acetic acid (AA) added, 'T' is the synthesis temperature, and 't' is the synthesis time.

S3.2. PXRD Data

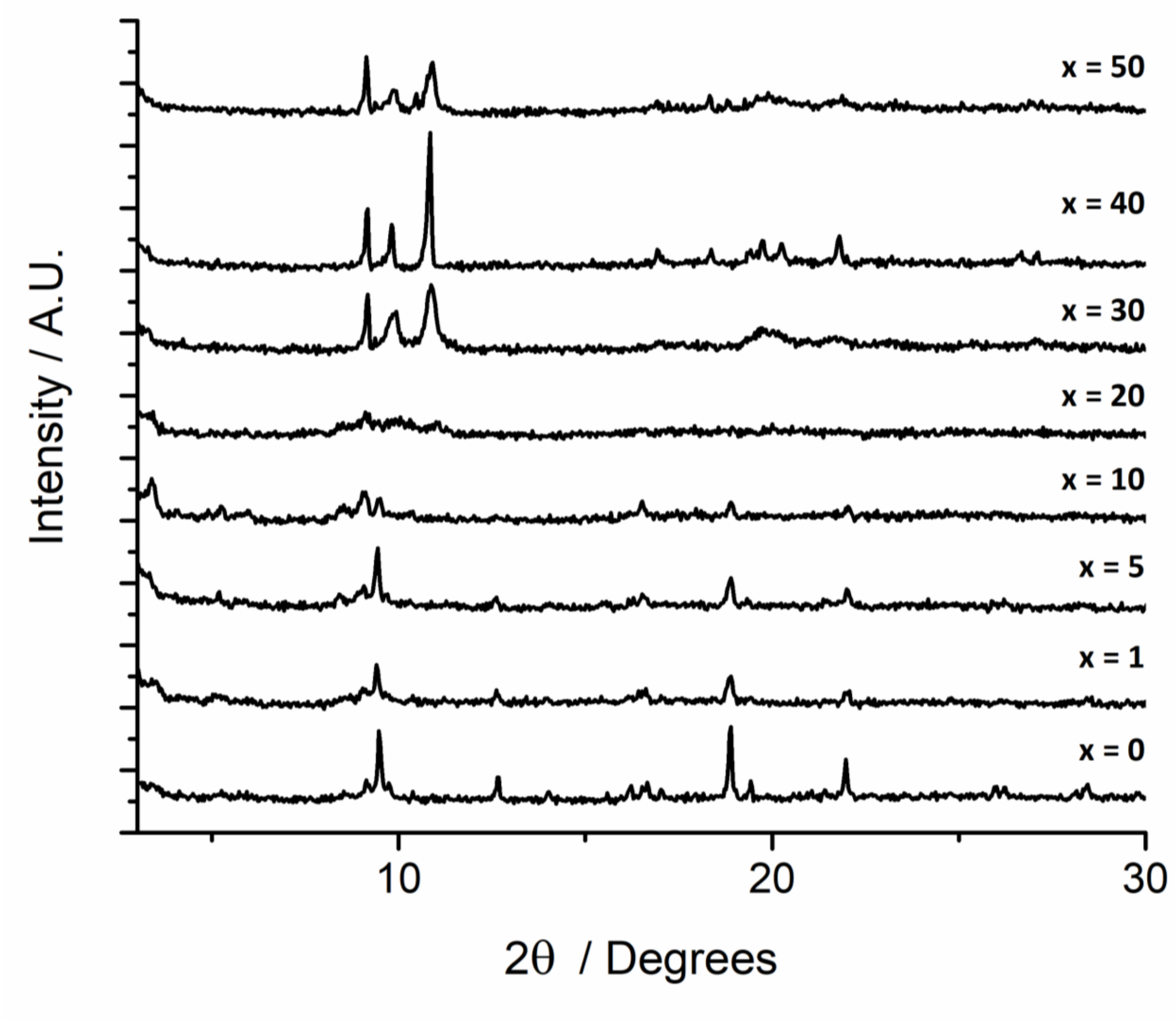

Figure S2. Stacked PXRD patterns of **$FeCl_3$-AAx(120°C,24h)** where '**x**' is the number of molar equivalents of acetic acid (**AA**) used in the synthesis. The main phases present are MOF-235(Fe) at low x and MIL-88B(Fe) and high x.

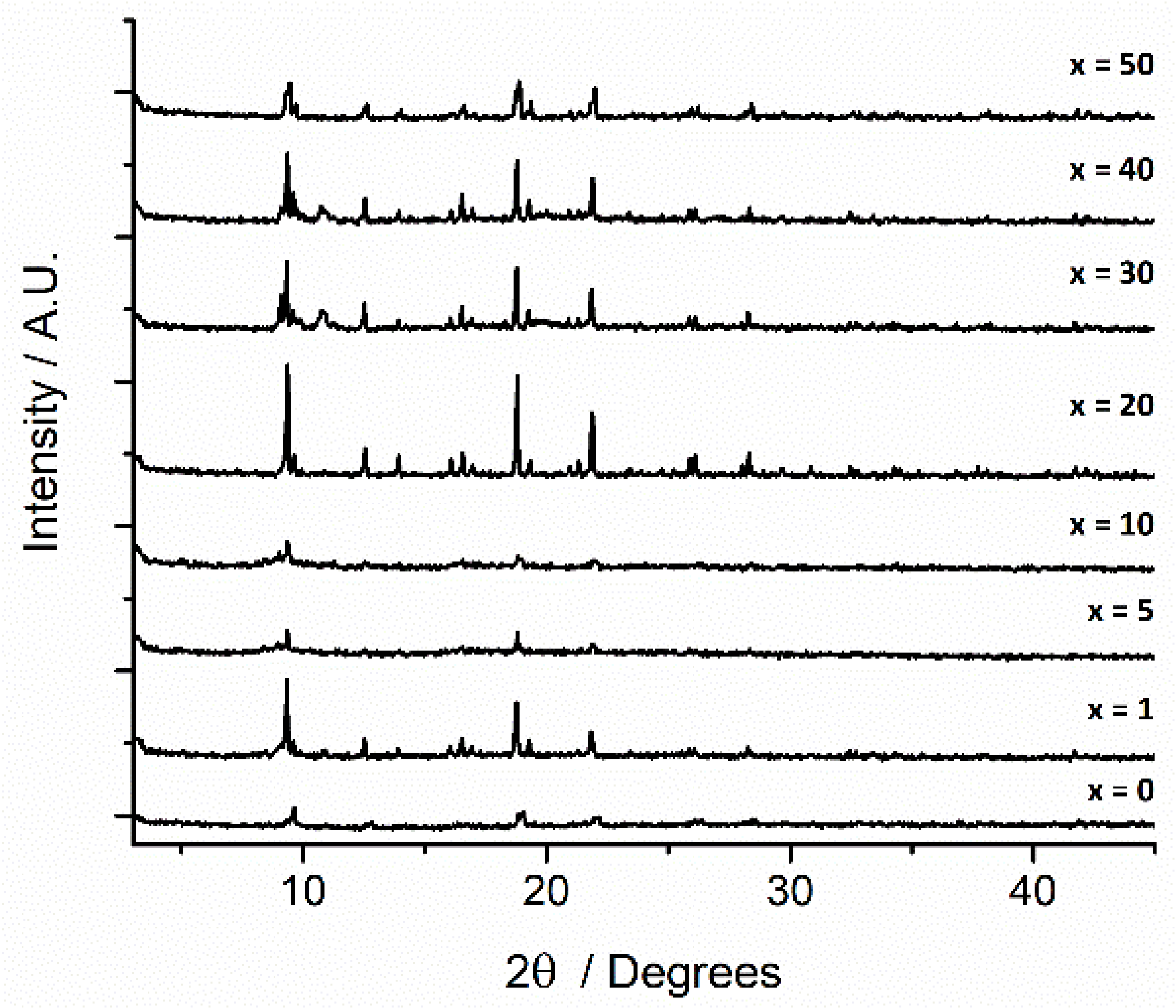

Figure S3. Stacked PXRD patterns of **$FeCl_3$-AAx(120°C,72h)**, where '**x**' is the number of molar equivalents of acetic acid (**AA**) used in the synthesis.

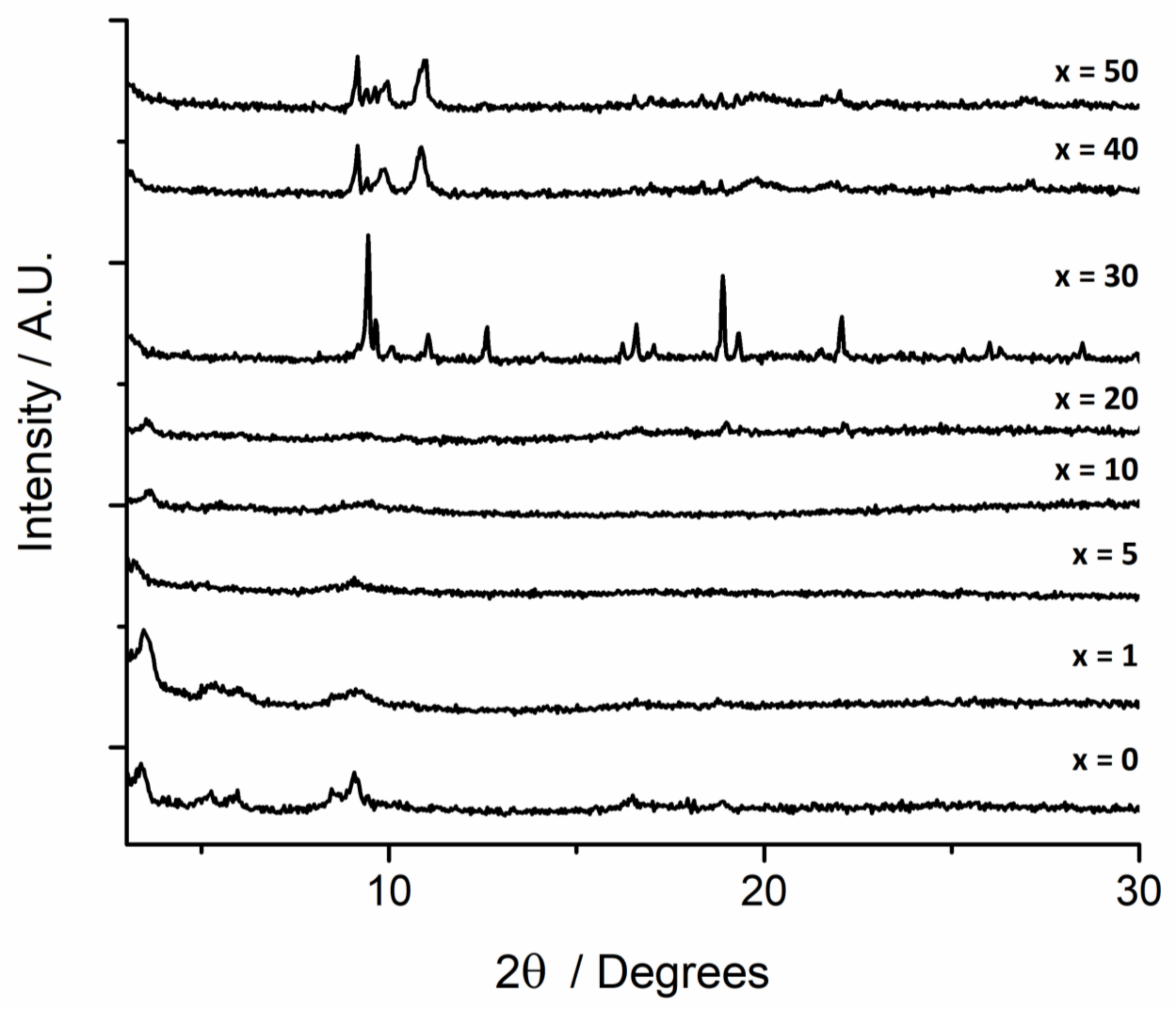

Figure S4. Stacked PXRD patterns of **$FeCl_2$-AAx(120°C,24h)** where '**x**' is the number of molar equivalents of acetic acid (**AA**) used in the synthesis. A minor phase/impurity is marked with an asterisk for x = 30.

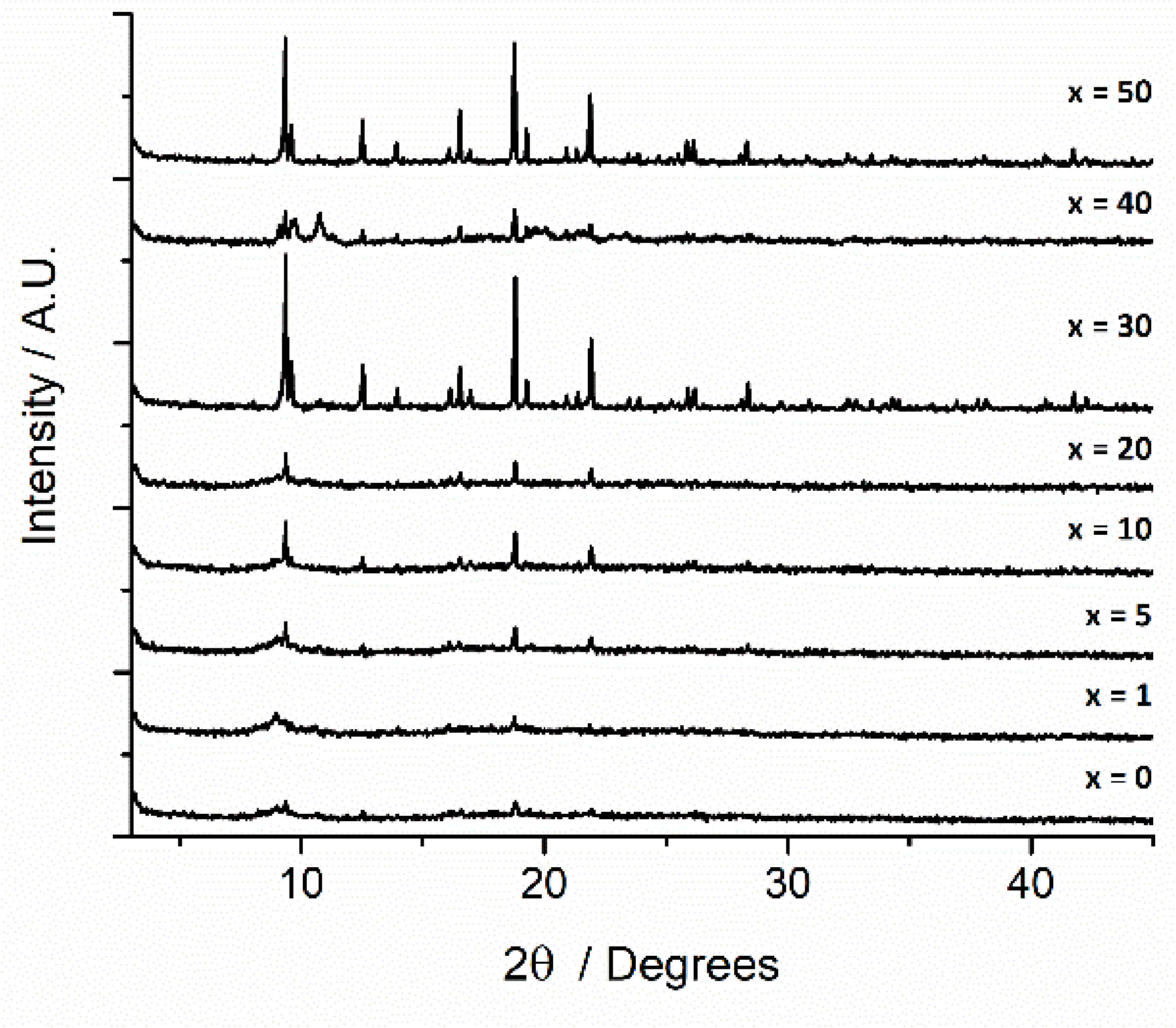

Figure S5. Stacked PXRD patterns of **$FeCl_2$-AAx(120°C,72h)**, where '**x**' is the number of molar equivalents of acetic acid (**AA**) used in the synthesis.

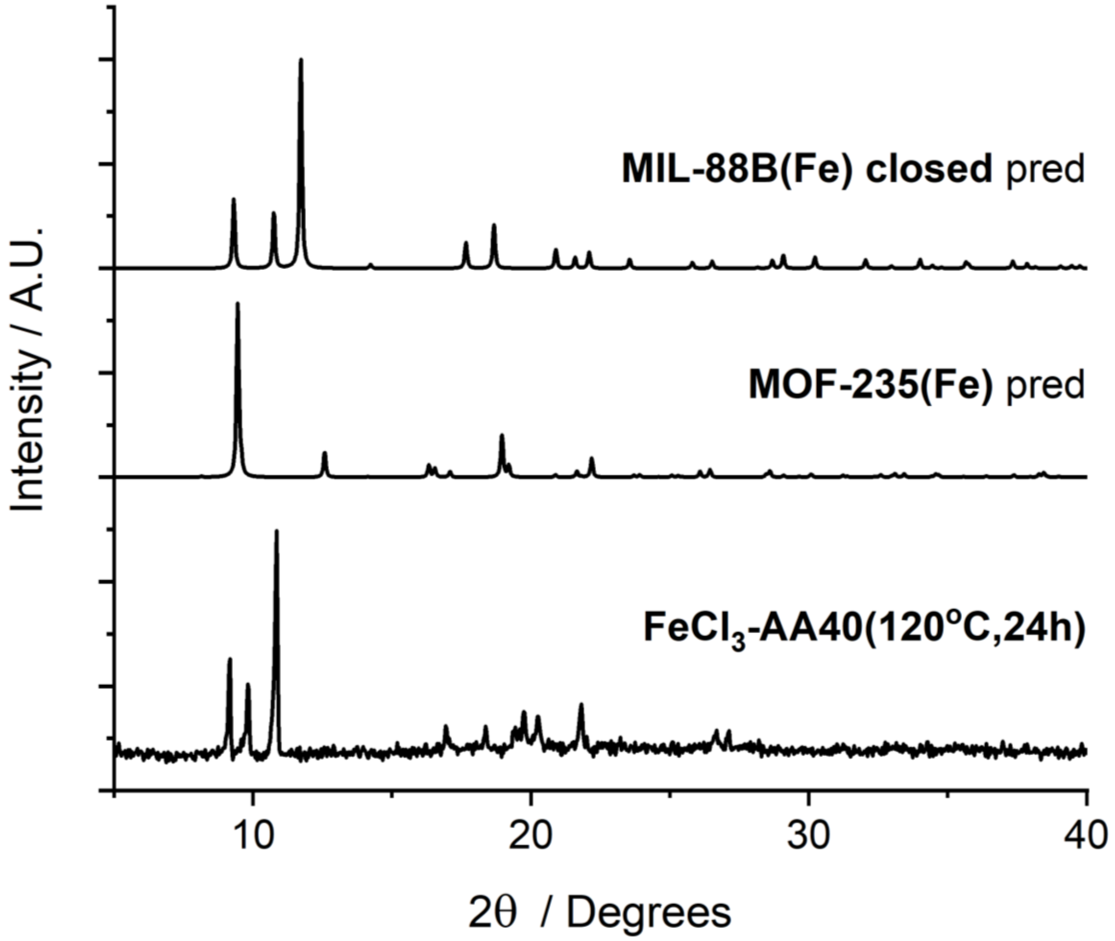

Figure S6. Stacked PXRD patterns of **$FeCl_3$-AA40(120°C,24h)** compared to the predicted patterns for MOF-235(Fe)[S6] and MIL-88B(Fe) closed, confirming the formation of MIL-88B(Fe).[S7] Some minor differences in the position of Bragg reflections are apparent, due to the sample not fully closing to the structure previously predicted. This phenomenon occurs throughout our characterisation of MIL-88B(Fe) samples, possibly due to adsorption of ambient moisture prior to measurement, but the presence of the three main Bragg reflections with characteristic intensities allows assignment of phase as MIL-88B(Fe).

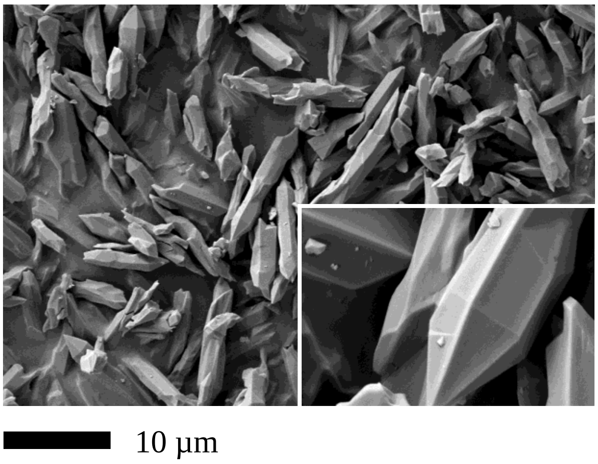

Figure S7. SEM image of **$FeCl_3$-AA40(120°C,24h)** showing characteristic MIL-88B pointed hexagonal rod morphology.

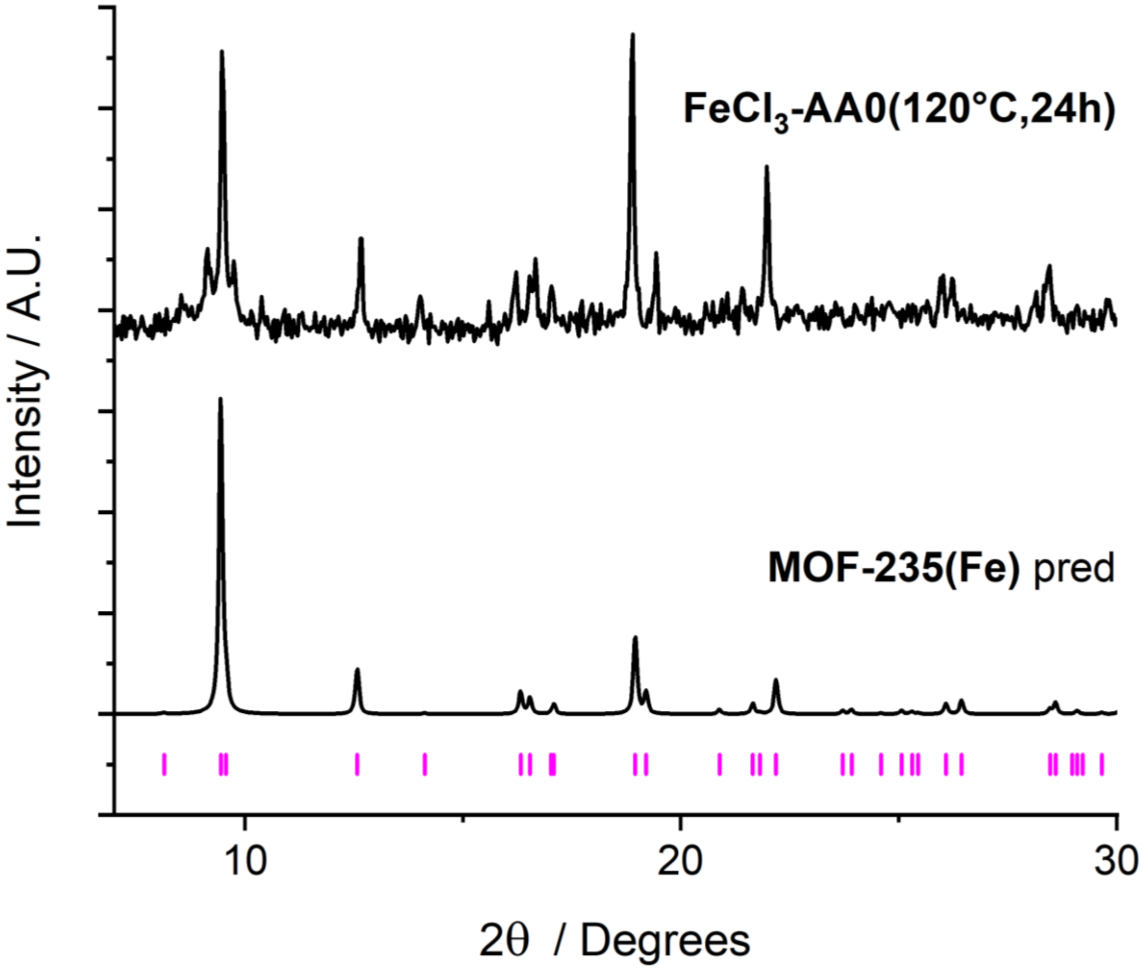

Figure S8. Comparison of the PXRD pattern of **$FeCl_3$-AA0(120°C,24h)** with the predicted pattern of MOF-235(Fe).[S6]

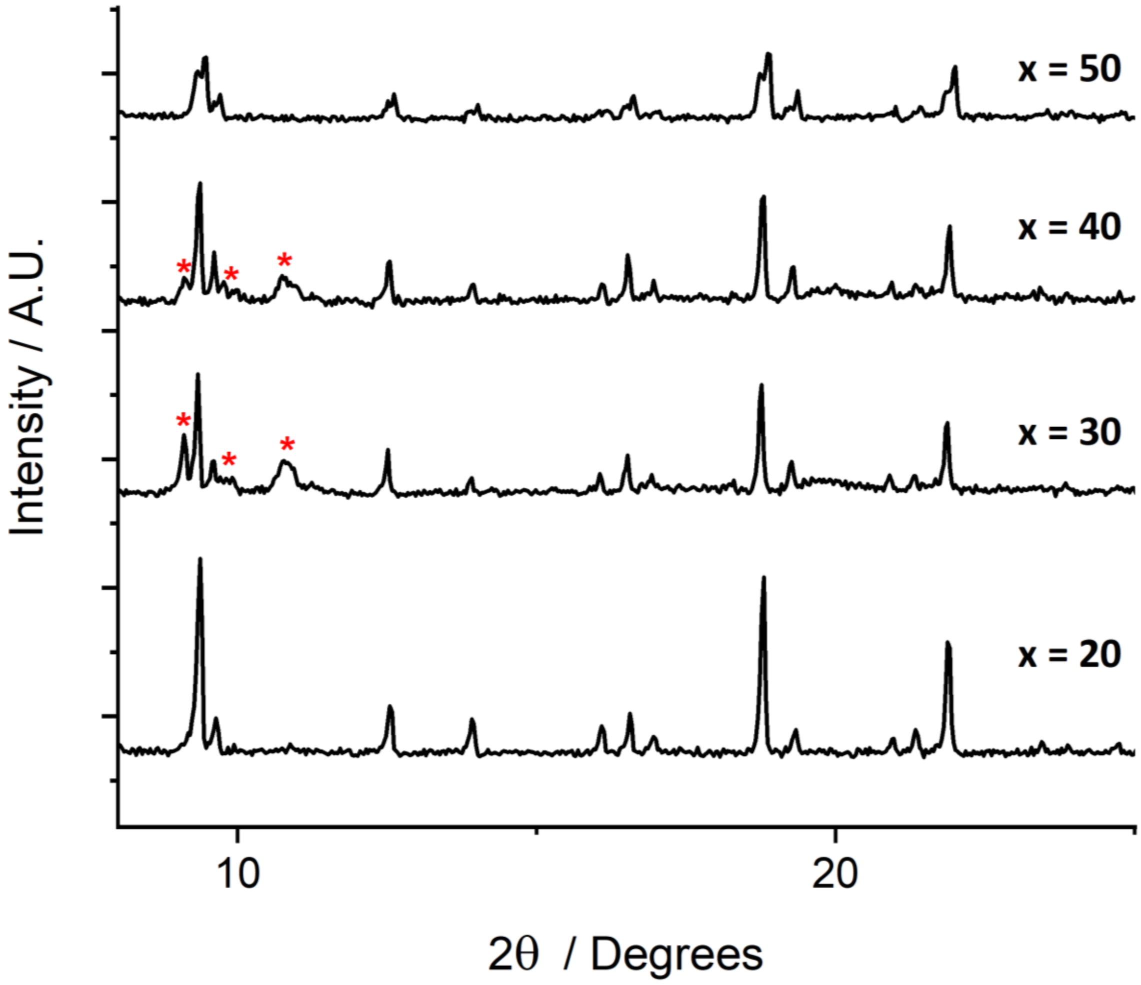

Figure S9. Stacked PXRD patterns of **$FeCl_3$-AAx(120°C,72h)** with 20-50 equivalents of acetic acid (**AA**) used in the synthesis (an enlargement from Figure S3), the Bragg peaks attributed to the MIL-88B(Fe) secondary phase are marked with red asterisks.

*

a)
b)
c)
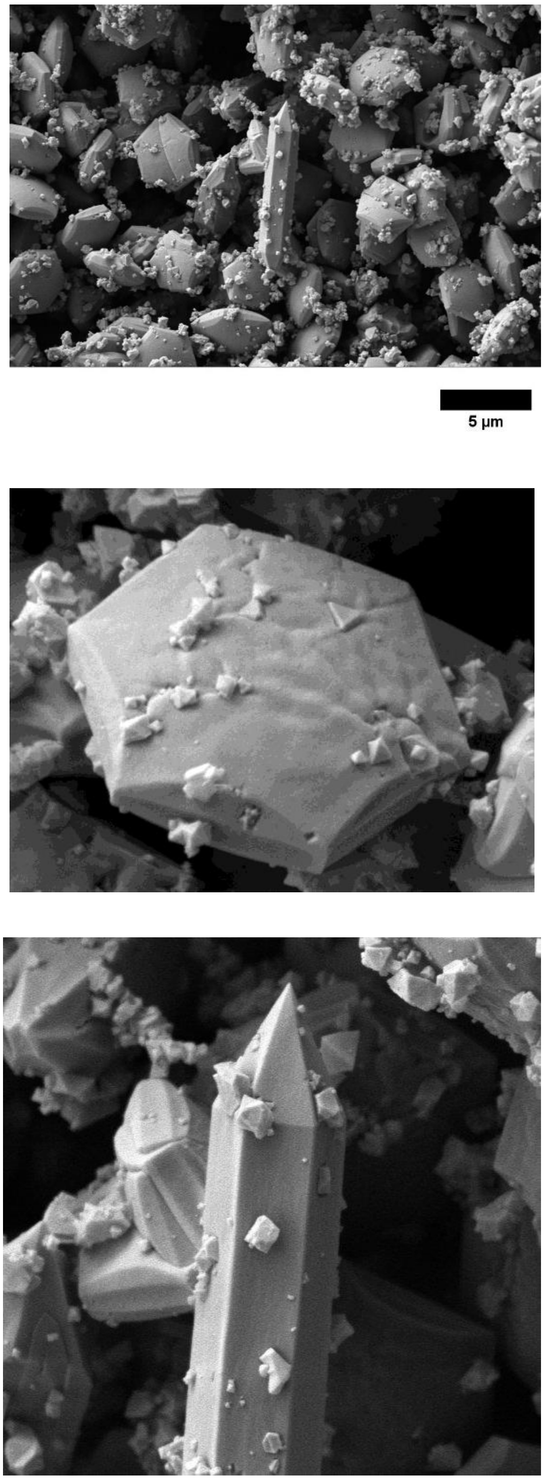

Figure S10. a) SEM image of **$FeCl_2$-AA30(120°C,24h)**, along with magnified images of crystals with morphologies corresponding to b) MOF-235, and c) MIL-88B(Fe).

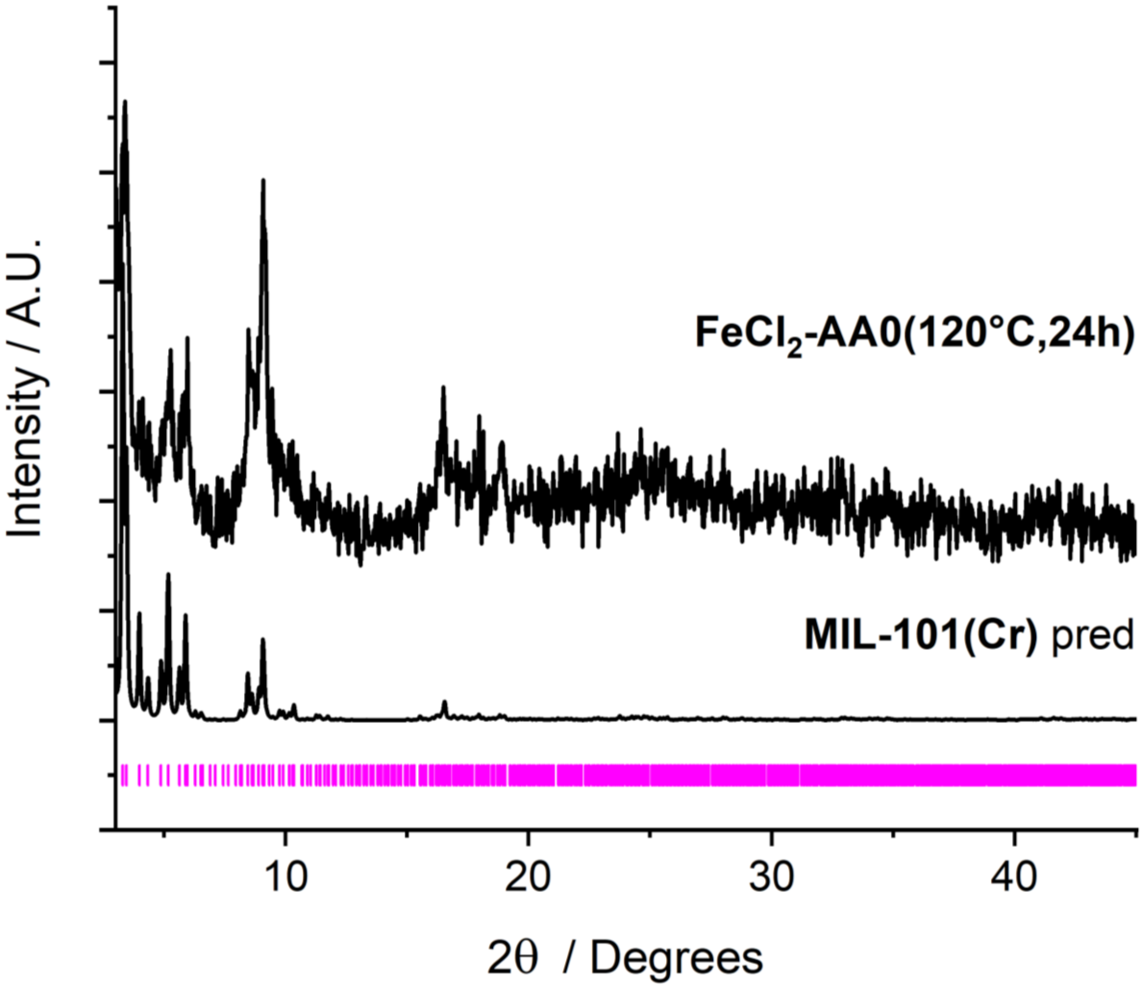

Figure S11. Comparison of the PXRD pattern of **$FeCl_2$-AA0(120°C,24h)** with the predicted pattern for MIL-101(Cr).[S4] The predicted PXRD pattern for MIL-101(Cr) is used, as there is no freely available data for MIL-101(Fe), and the two MOFs are isostructural.

S.4. Synthesis with Variable Heating Time

S4.1. General Conditions for Synthesis

Either iron(II) chloride tetrahydrate or iron(III) chloride hexahydrate (1 mmol) and terephthalic acid (1 mmol) were added to a 50 mL Pyrex reagent jar and DMF (10 mL) was added; for modulated samples acetic acid (30 mmol) was also added. The jar was capped and sonicated until the solids dissolved, before heating in an isothermal oven at either 120 °C or 150 °C for either 2, 4, 24, 48, 72, 120 or 168 hours. After allowing to cool to room temperature, the suspension was separated by centrifuging. The supernatant was decanted and fresh DMF (20 mL) was added before centrifuging again. This was repeated three times, before repeating the procedure another three times with DCM (20 mL). The sample was then dried overnight in a desiccator under vacuum. The naming system for these samples is **$FeCl_2$-AAx(T,t)** and **$FeCl_3$-AAx(T,t)**, where ‘x’ equals the number of molar equivalents of acetic acid (AA) added, ‘T’ is the synthesis temperature, and ‘t’ is the synthesis time.

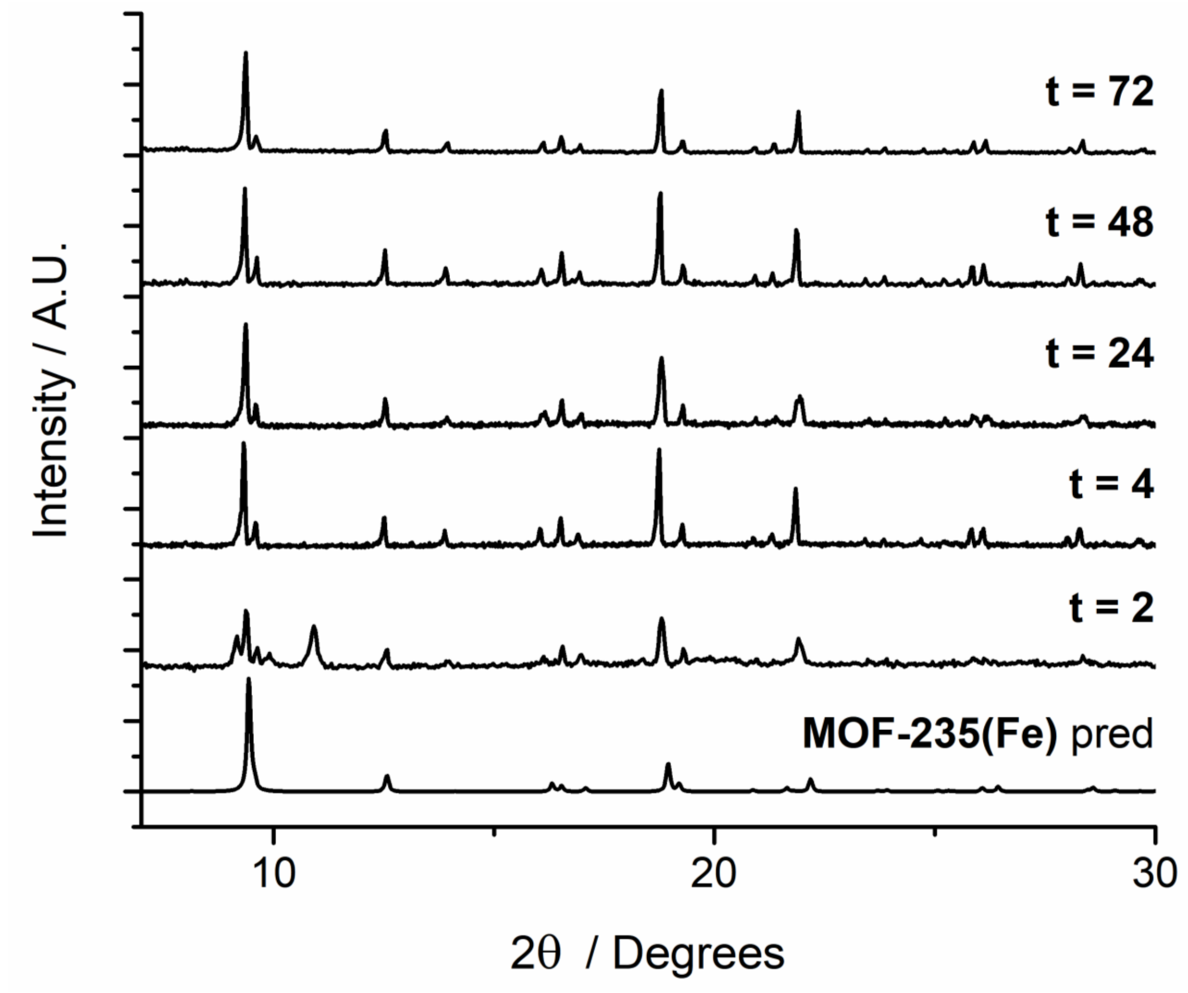

Figure S12. Stacked PXRD patterns of **$FeCl_3$-AA30(150°C,t)**, where '**t**' is the synthesis time, compared to the predicted pattern for MOF-235(Fe).[S6] A minor MIL-88B(Fe) impurity is visible for **$FeCl_3$-AA30(150°C,2)**.

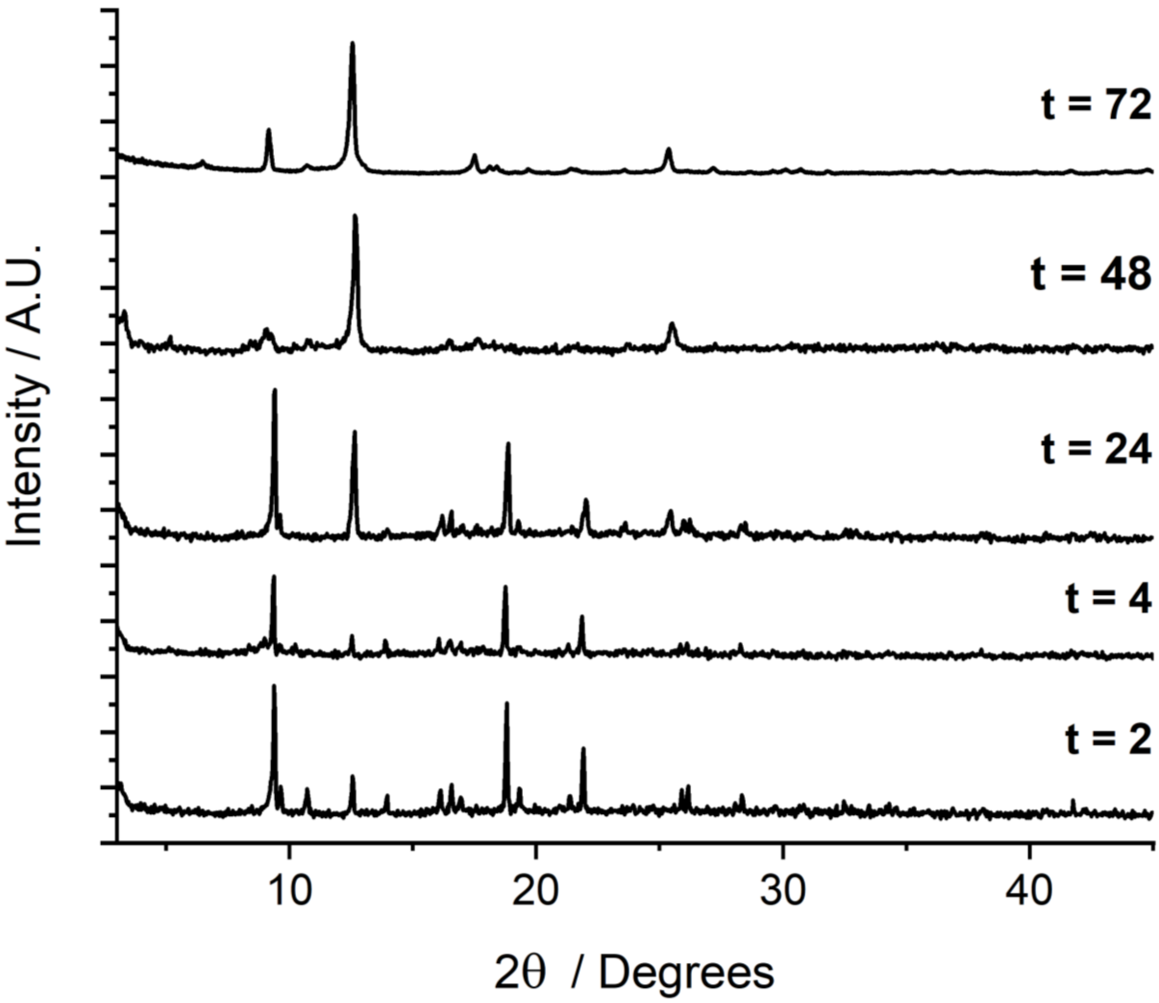

Figure S13. Stacked PXRD patterns of **$FeCl_3$-AA0(150°C,t)**, where '**t**' is the synthesis time.

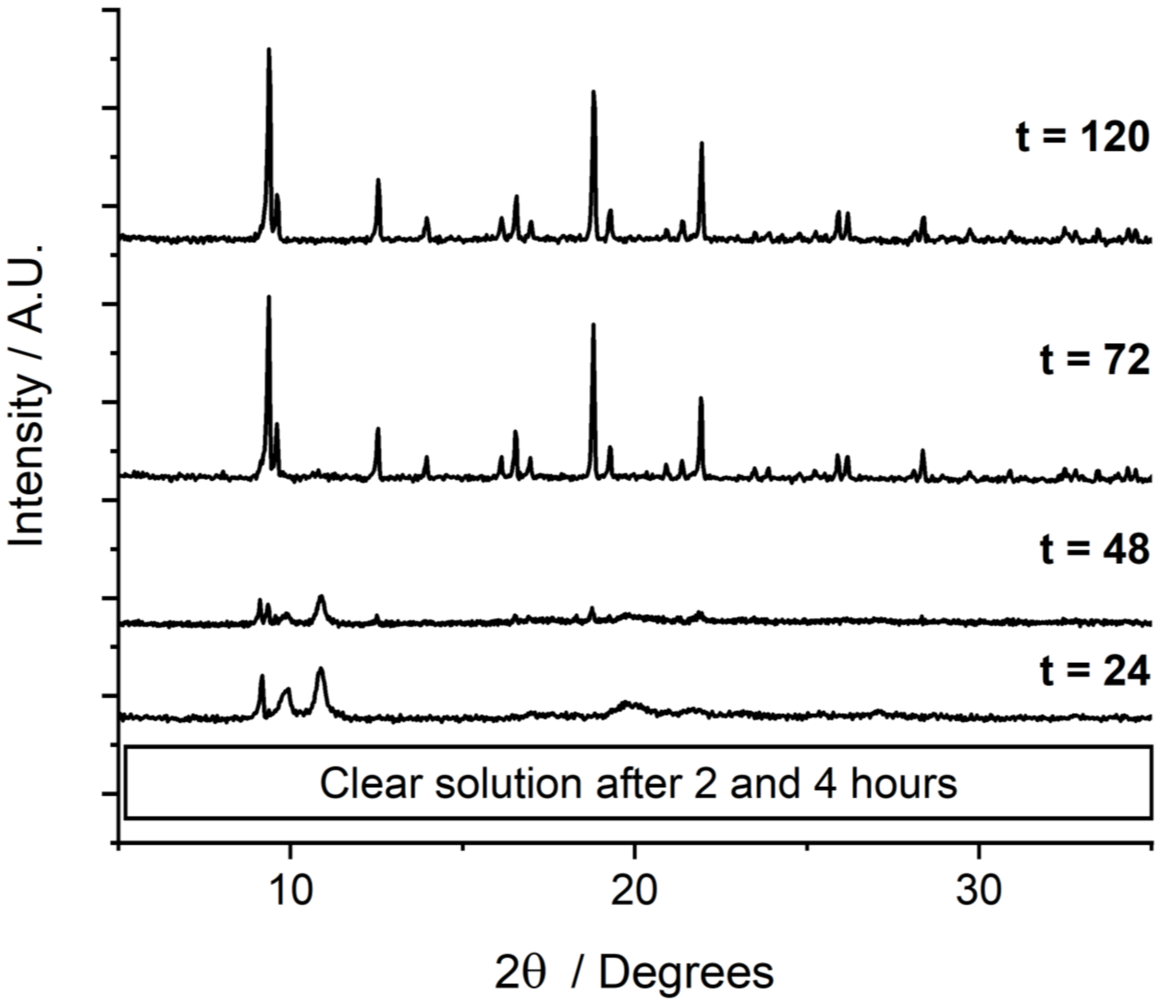

Figure S14. Stacked PXRD patterns of **$FeCl_3$-AA30(120°C,t)**, where '**t**' is the synthesis time.

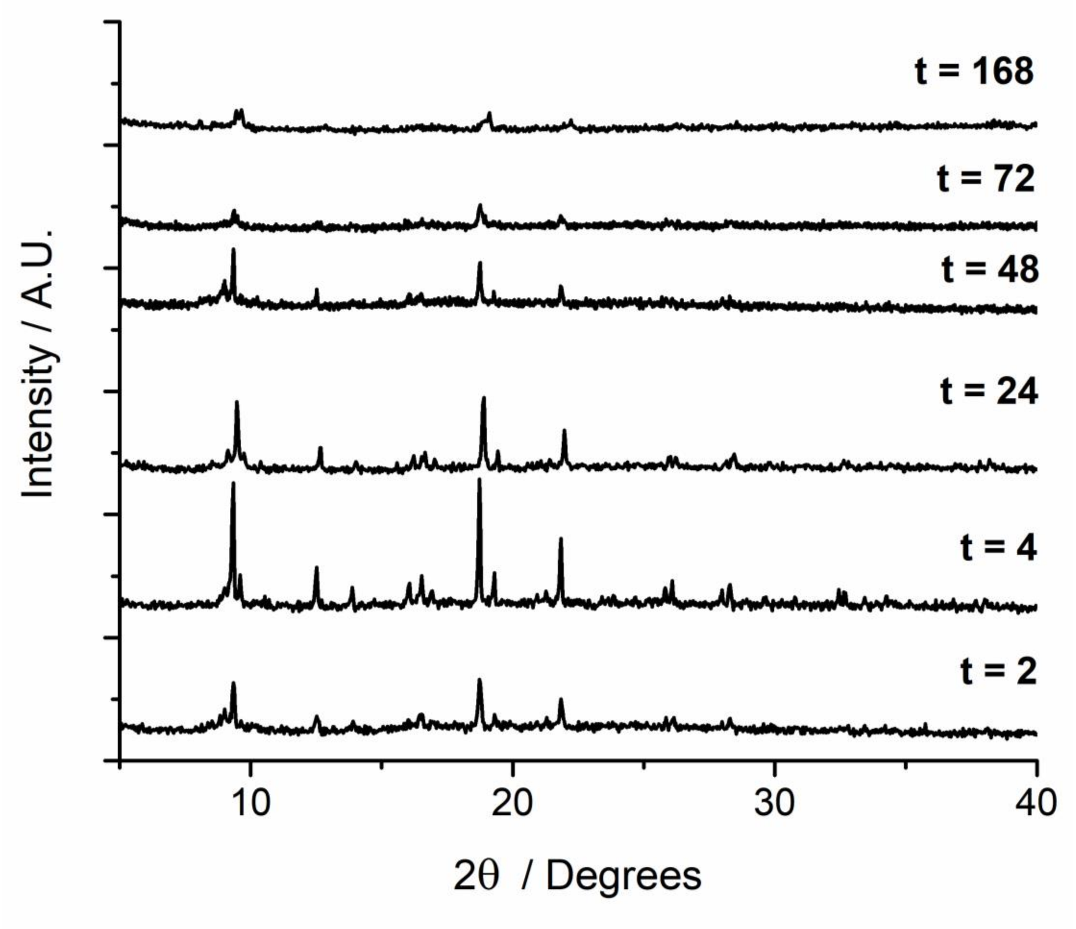

Figure S15. Stacked PXRD patterns of **$FeCl_3$-AA0(120°C,t)**, where '**t**' is the synthesis time.

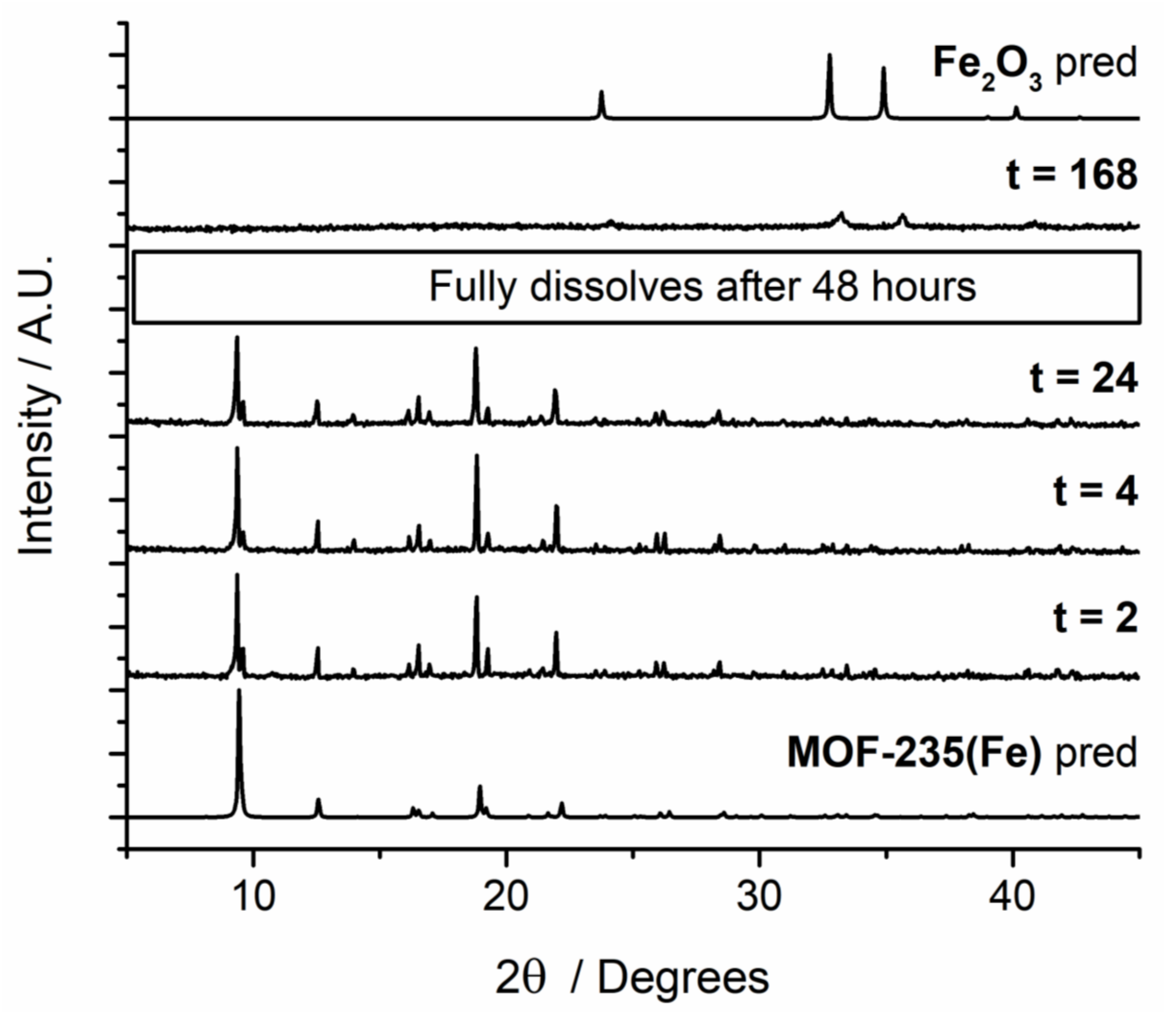

Figure S16. Stacked PXRD patterns of **$FeCl_2$-AA30(150°C,t)**, where '**t**' is the synthesis time, compared to the predicted patterns for MOF-235(Fe)[S6] and α-Fe_2O_3 (hematite).[S8]

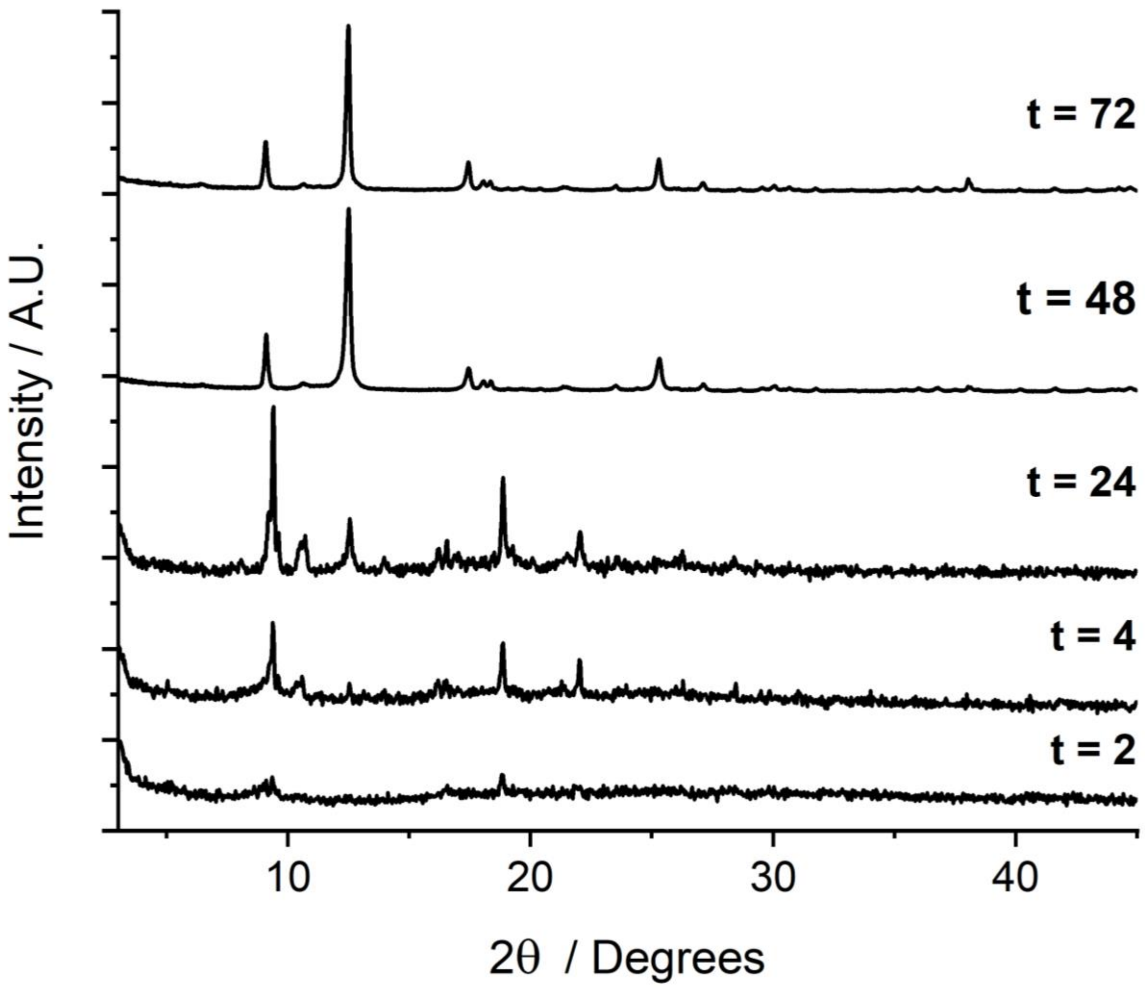

Figure S17. Stacked PXRD patterns of **$FeCl_2$-AA0(150°C,t)**, where '**t**' is the synthesis time. On drying from DCM, MIL-53(Fe) is present as the MIL-53(Fe)_lt phase at t = 48 and 72 h (see Figure S25).

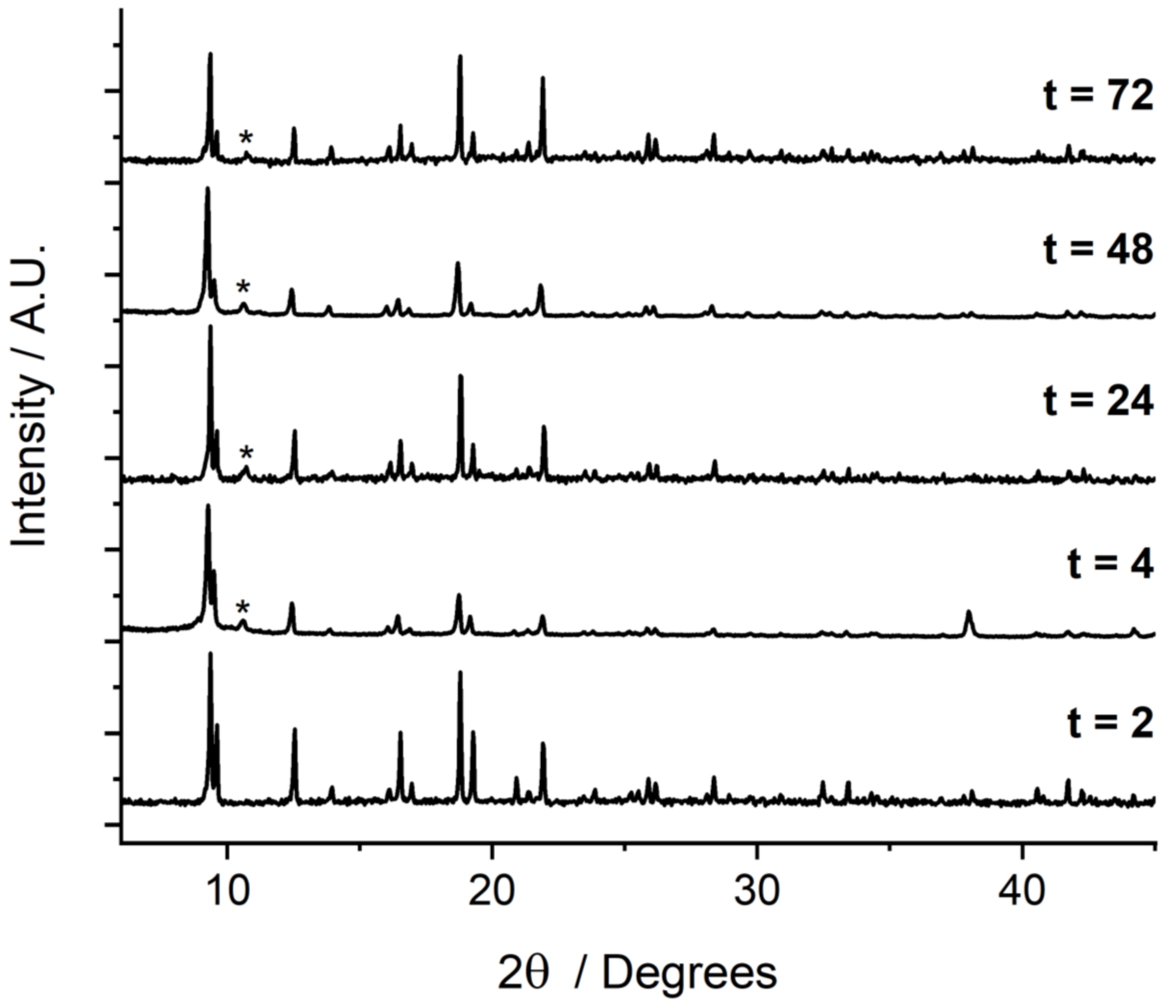

Figure S18. Stacked PXRD patterns of **$FeCl_2$-AA30(120°C,t)**, where '**t**' is the synthesis time. The minor phase/impurity is marked with asterisks.

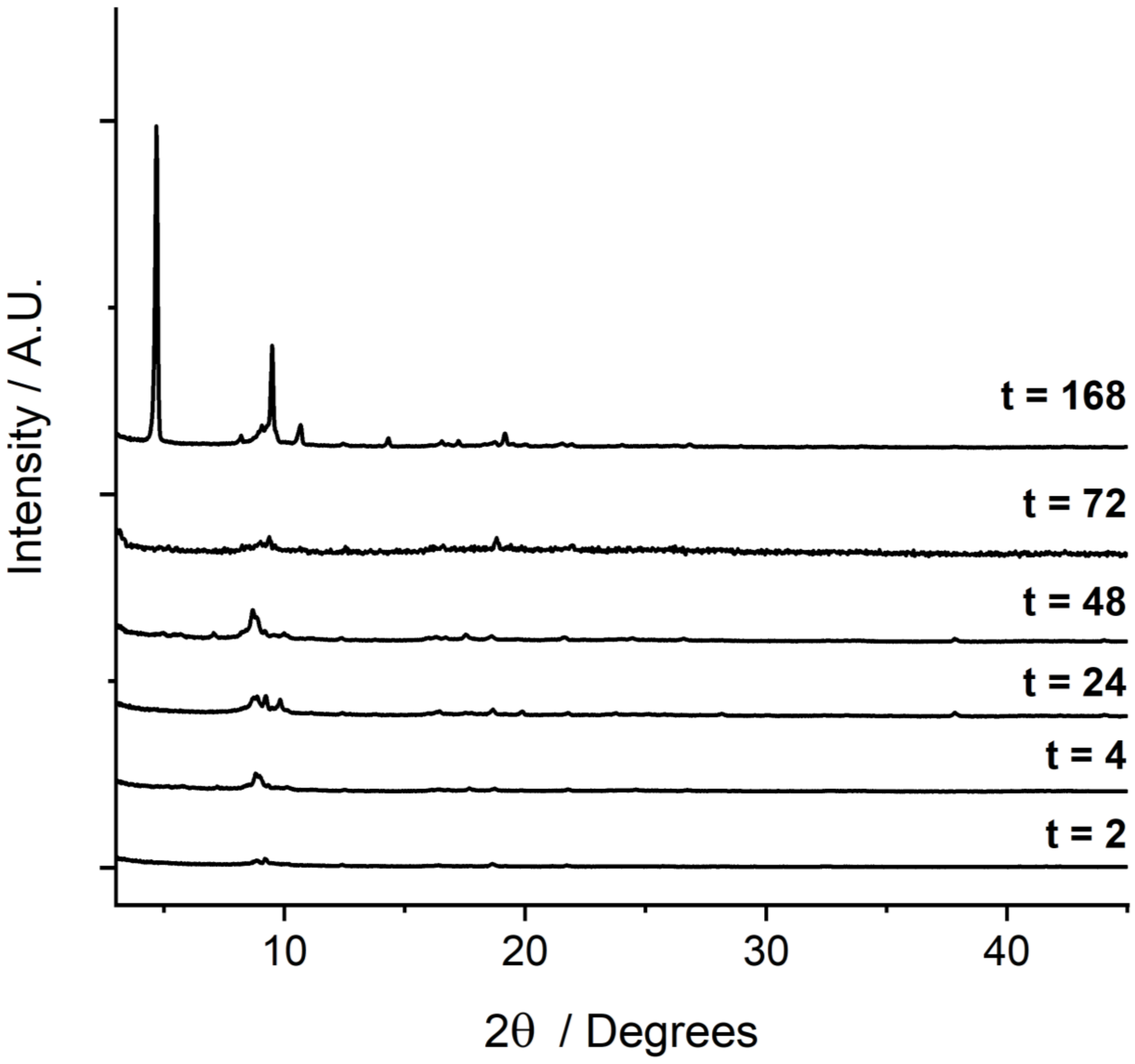

Figure S19. Stacked PXRD patterns of **$FeCl_2$-AA0(120°C,t)**, where ‘**t**’ is the synthesis time.

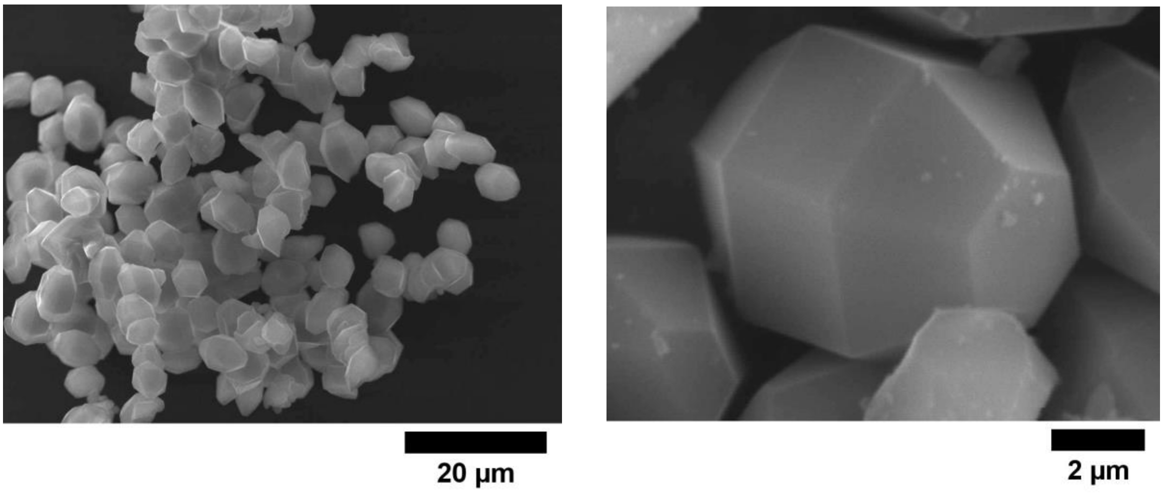

Figure S20. SEM images of **$FeCl_3$-AA30(150°C,72h)**. The truncated morphology differs from the pointed hexagonal prisms of MIL-88B(Fe).

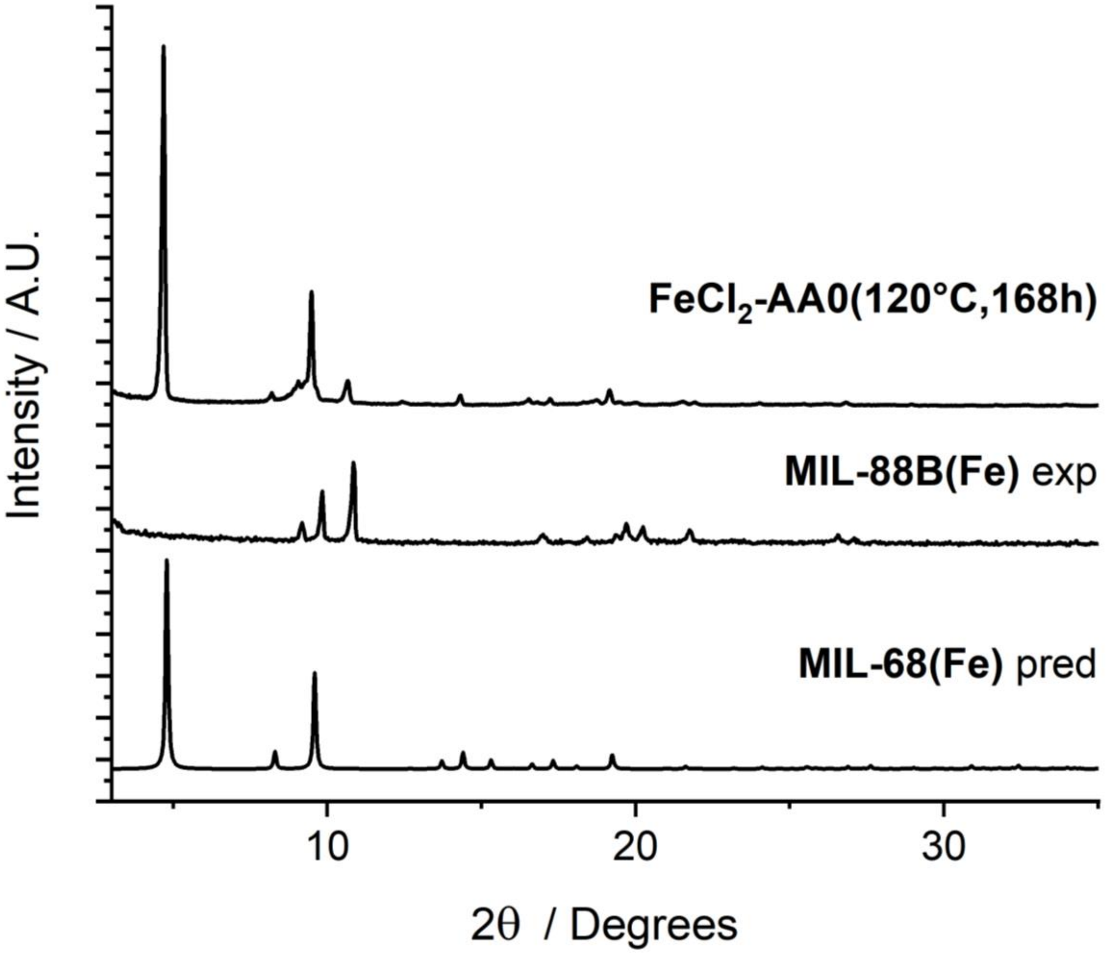

Figure S21. Stacked PXRD patterns of **$FeCl_2$-AA0(120°C,168h)**, compared to the predicted pattern for MIL-68(Fe)[S5] and an experimental pattern for MIL-88(Fe) (dry state).

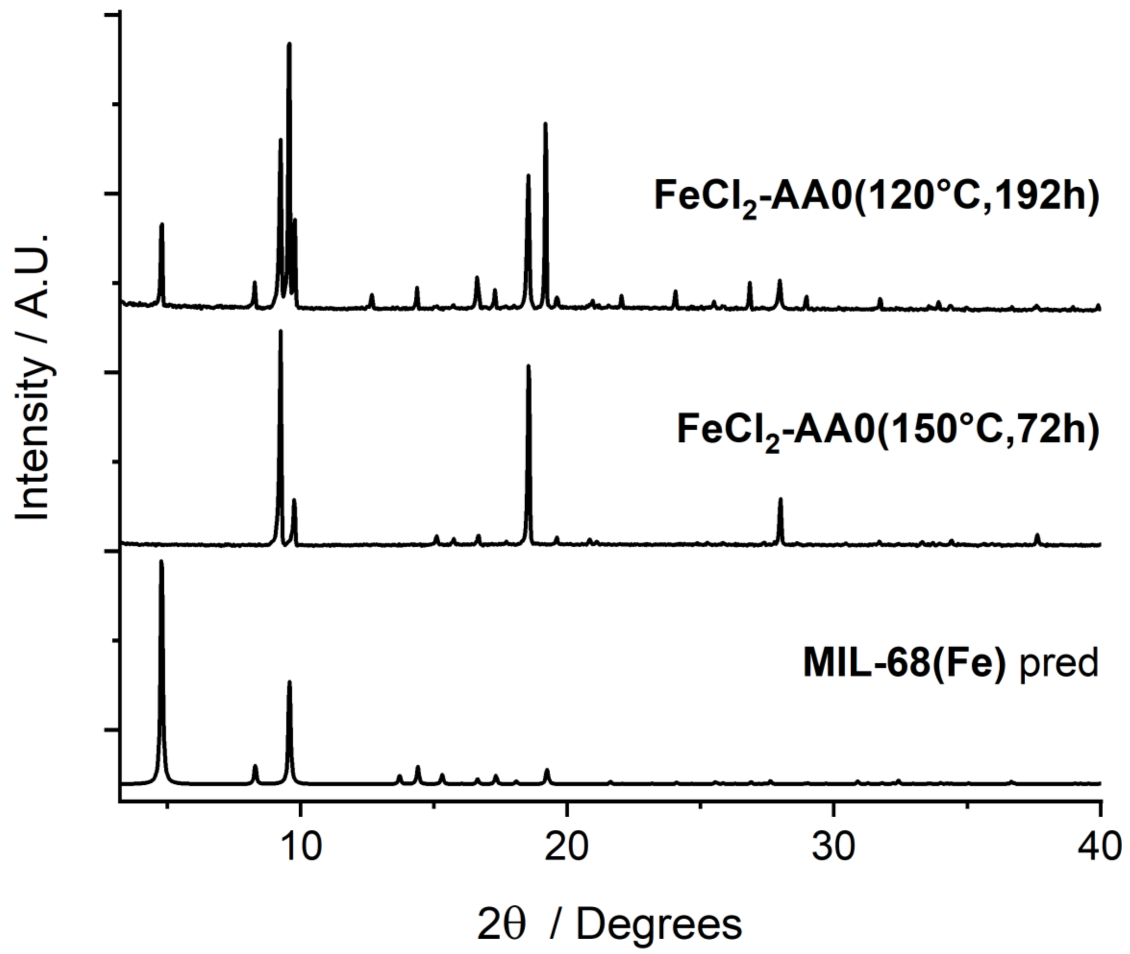

Figure S22. Stacked PXRD patterns of **$FeCl_2$-AA0(120°C,192h)**, compared to the predicted pattern for MIL-68(Fe)[S5] and an experimental pattern for MIL-53(Fe)-DMF (**$FeCl_2$-AA0(150°C,72h)**).

S4.2. Single-Crystal Synthesis of MIL-53(Fe)-DMF

$FeCl_2 \cdot 4H_2O$ (1 mmol) and terephthalic acid (1 mmol) were added to a 50 mL Pyrex reagent jar and DMF (10 mL) was added. The jar was capped and sonicated until the solids dissolved, before heating at 150 ˚C in an isothermal oven. After 3 days, the jar was removed and allowed to cool to room temperature. Yellow needle-shaped crystals were evident and the DMF was decanted and replaced several times with fresh DMF, in which the crystals were kept for further analysis. A formula of [Fe(OH)(BDC)]·DMF was determined by elemental analysis and TGA. CHN. Expected ($FeC_8H_5O_5 \cdot C_3H_7NO$): C, 42.60; H, 3.87; N, 4.52. Found: C, 42.36; H, 3.83; N, 4.83. Two mass losses can be seen by TGA (Figure S23): one up to 200 ˚C due to solvent loss, and another which starts around 300 ˚C corresponding to framework degradation. 1st mass loss = 21.4% (DMF, theoretical = 23.6%), 2nd mass loss = 48.2% (BDC, theoretical = 50.7%).

Crystal data for MIL-53(Fe)-DMF: $C_8H_5FeO_5$, M_r = 236.97, crystal dimensions 0.16 × 0.04 × 0.03 mm, Triclinic, a = 6.8403 (12) Å, b = 10.7142 (18) Å, c = 10.7928 (18) Å, V = 669.5 (2) Å^3, T = 150 K, space group $P\bar{1}$, (No. 2), Z = 2, 13960 measured reflections, 3285 independent reflections (R_{int} = 0.054), which were used in all calculations. The final R_1 = 0.060 for 2479 observed data $R[F^2 > 2\sigma(F^2)]$ and $wR(F^2)$ = 0.137 (all data). CCDC Deposition 2088536.

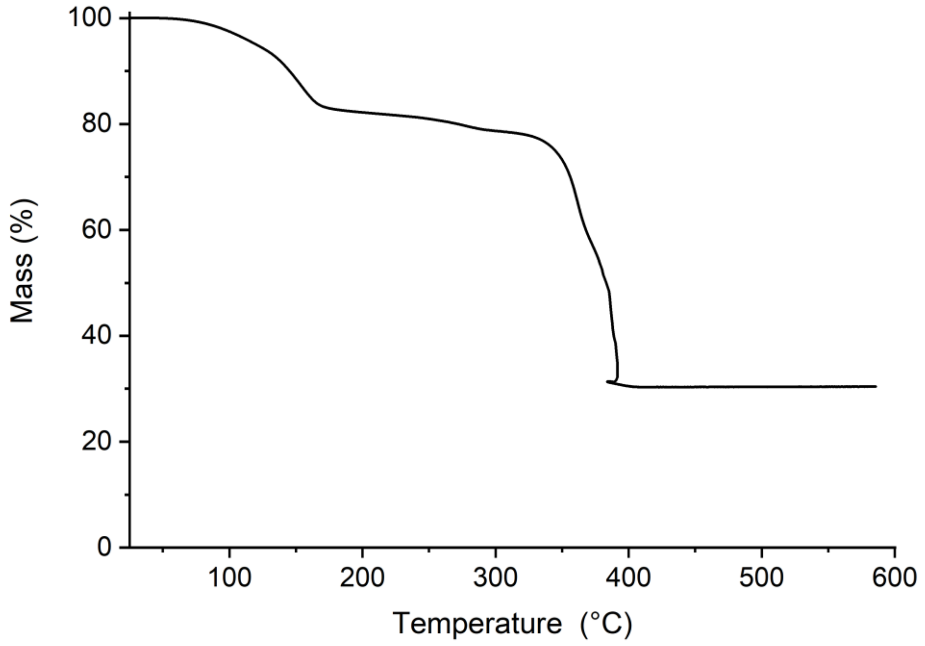

Figure S23. TGA profile for MIL-53(Fe)-DMF.

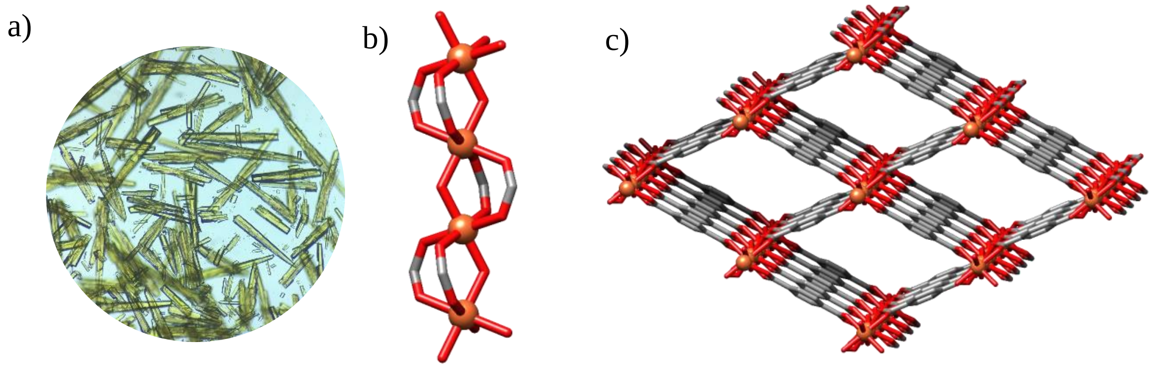

Figure S24. a) Image of crystals of MIL-53(Fe)-DMF, b) Chain SBU present in MIL-53(Fe)-DMF, c) Projection of the crystal structure of MIL-53(Fe)-DMF at a slight angle from the crystallographic a axis.

S4.3. PXRD Data

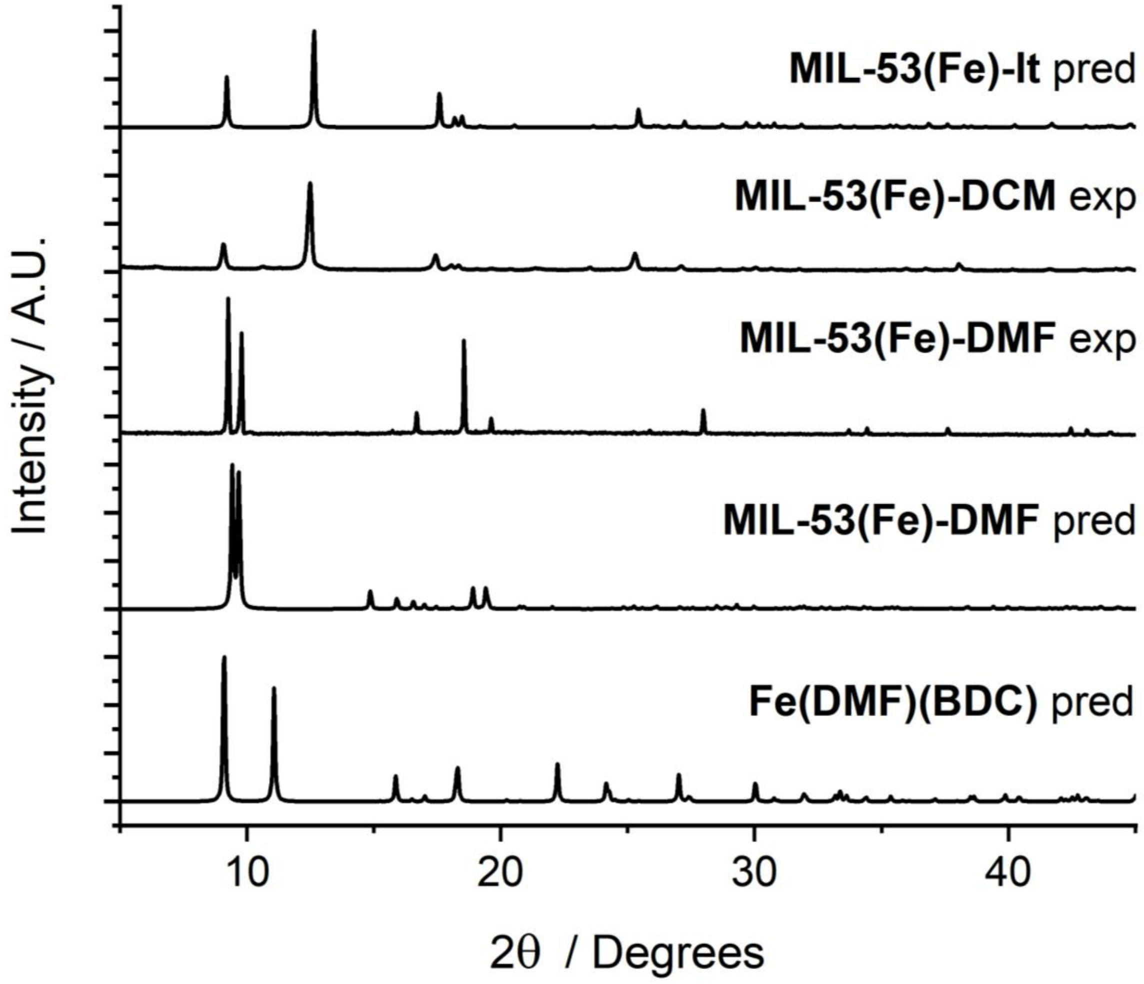

Figure S25. Stacked experimental (exp) PXRD patterns of MIL-53(Fe)-DMF (air dried from DMF) and MIL-53(Fe)-DCM (solvent exchange with DCM and air dried), compared with the predicted (pred) PXRD patterns of Fe(DMF)(BDC), MIL-53(Fe)-DMF, and MIL-53(Fe)-lt (the hydrated phase of MIL-53(Fe)).[S3]

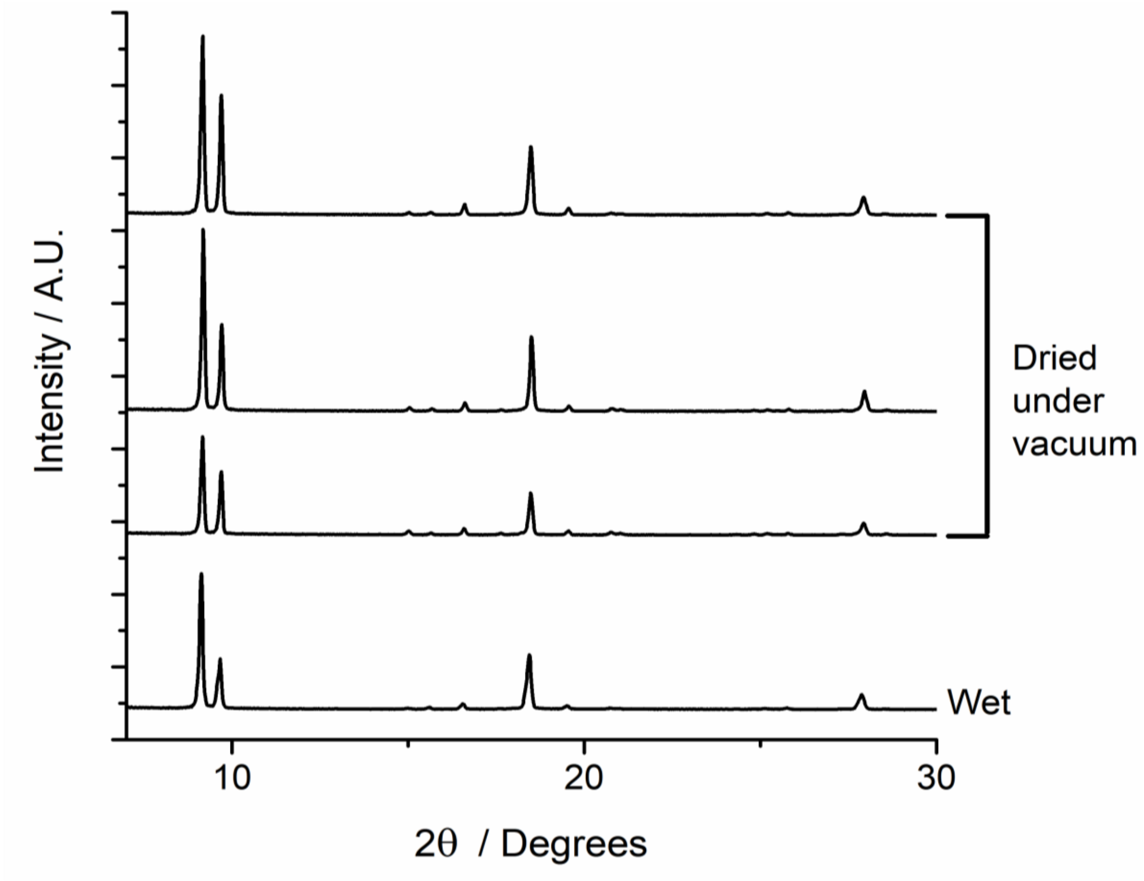

Figure S26. Stacked PXRD patterns of MIL-53(Fe)-DMF taken wet and after drying overnight under vacuum at RT (no DCM wash) showing that DMF is retained.

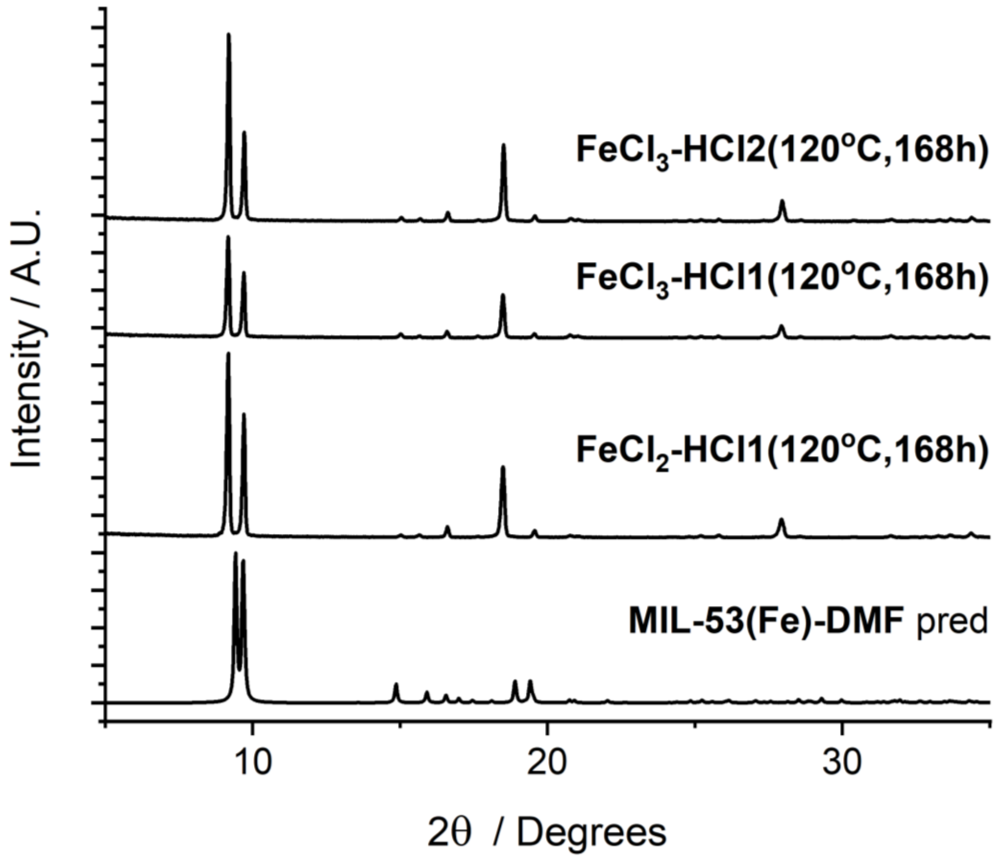

Figure S27. Stacked PXRD patterns of Fe-BDC synthesised at 120 °C for 168 hours with HCl (1 or 2 molar equivalents) as modulator, using either $FeCl_2·4H_2O$ or $FeCl_3·6H_2O$ as starting material, compared to the predicted PXRD pattern of MIL-53(Fe).

S.5. Synthesis with Varying Fe-precursor

S5.1. General Conditions for Synthesis

The iron precursor (1 mmol) and terephthalic acid (1 mmol) were added to a 50 mL Pyrex reagent jar and DMF (10 mL) was added, for modulated samples acetic acid (1-50 mmols) was also added. The jar was capped and sonicated until the solids dissolved before heating in an isothermal oven at 120 °C for either 24 or 72 hours. After allowing to cool to room temperature, the suspension was separated by centrifuging. The supernatant was decanted and fresh DMF (20 mL) was added before centrifuging again. This was repeated three times, before repeating the procedure another three times with DCM (20 mL). The sample was then dried overnight in a desiccator under vacuum. The naming system used is **Fe(counterion)-AA**x(T,t) where 'x' equals the number of molar equivalents of acetic acid (**AA**) added, 'T' is the synthesis temperature, and 't' is the synthesis time.

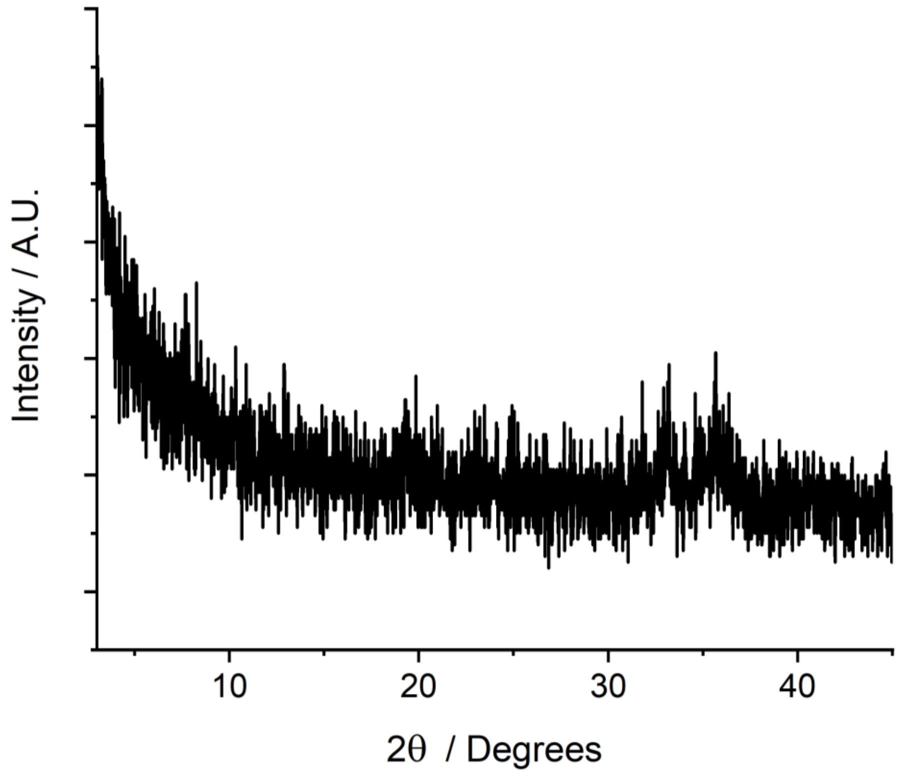

Figure S28. PXRD pattern of **Fe(NO$_3$)$_3$-AA0(150°C,72h)**.

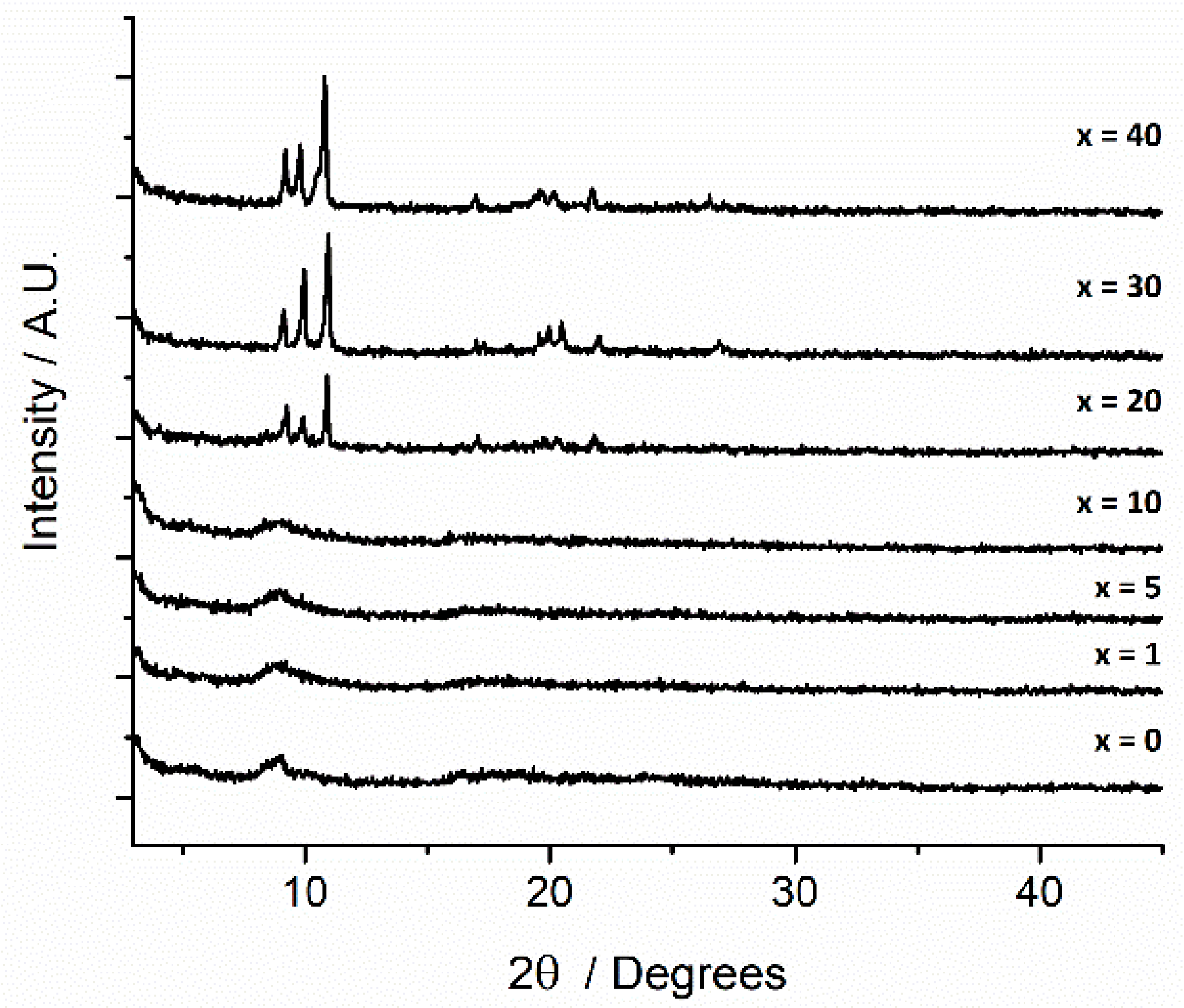

Figure S29. Stacked PXRD patterns of a) **$Fe(NO_3)_3$-AAx(120°C,24h)**, where '**x**' is the number of molar equivalents of acetic acid (**AA**) used in the synthesis.

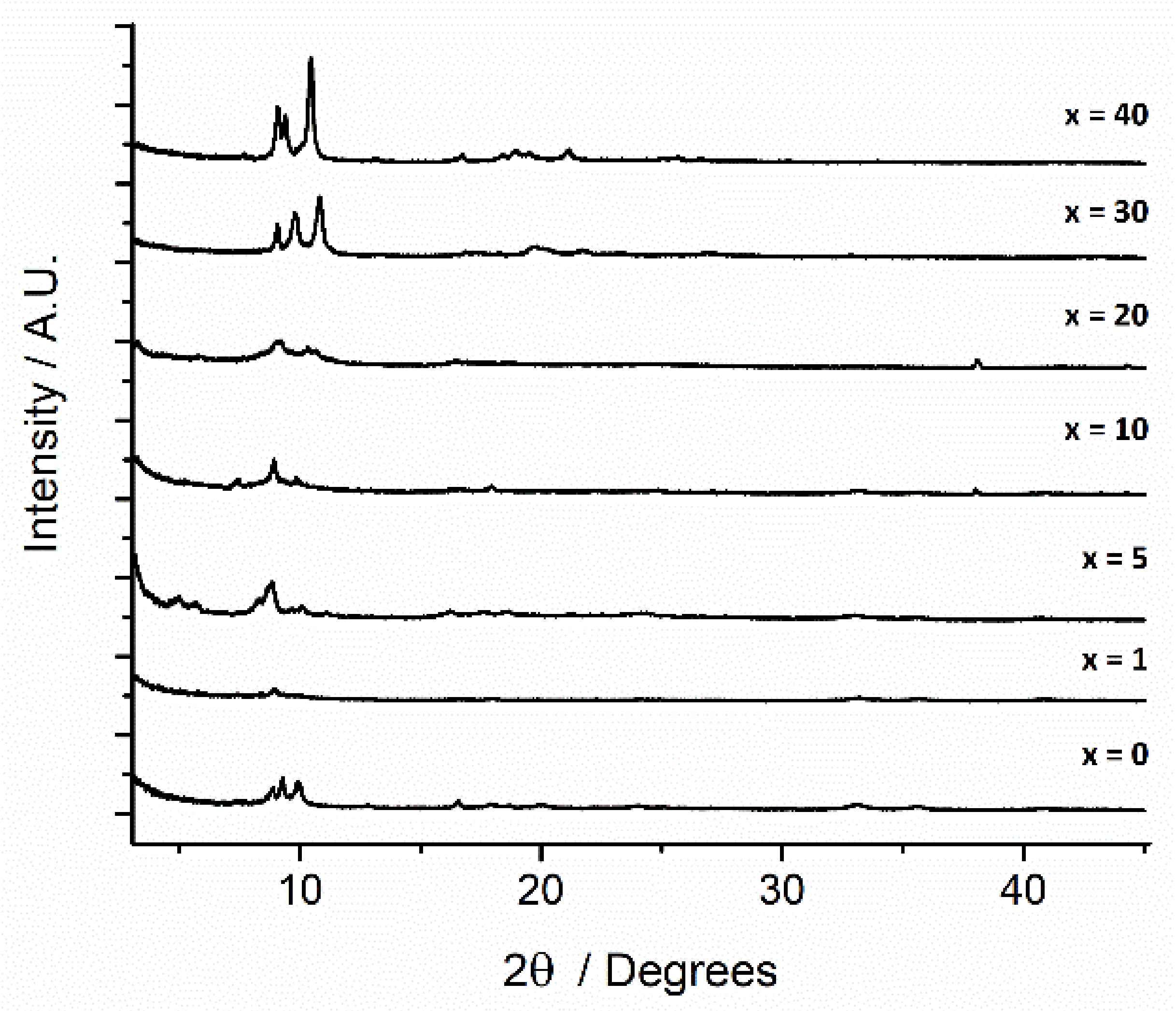

Figure S30. Stacked PXRD patterns of **$Fe(NO_3)_3$-AAx(120°C,72h)**, where '**x**' is the number of molar equivalents of acetic acid (**AA**) used in the synthesis.

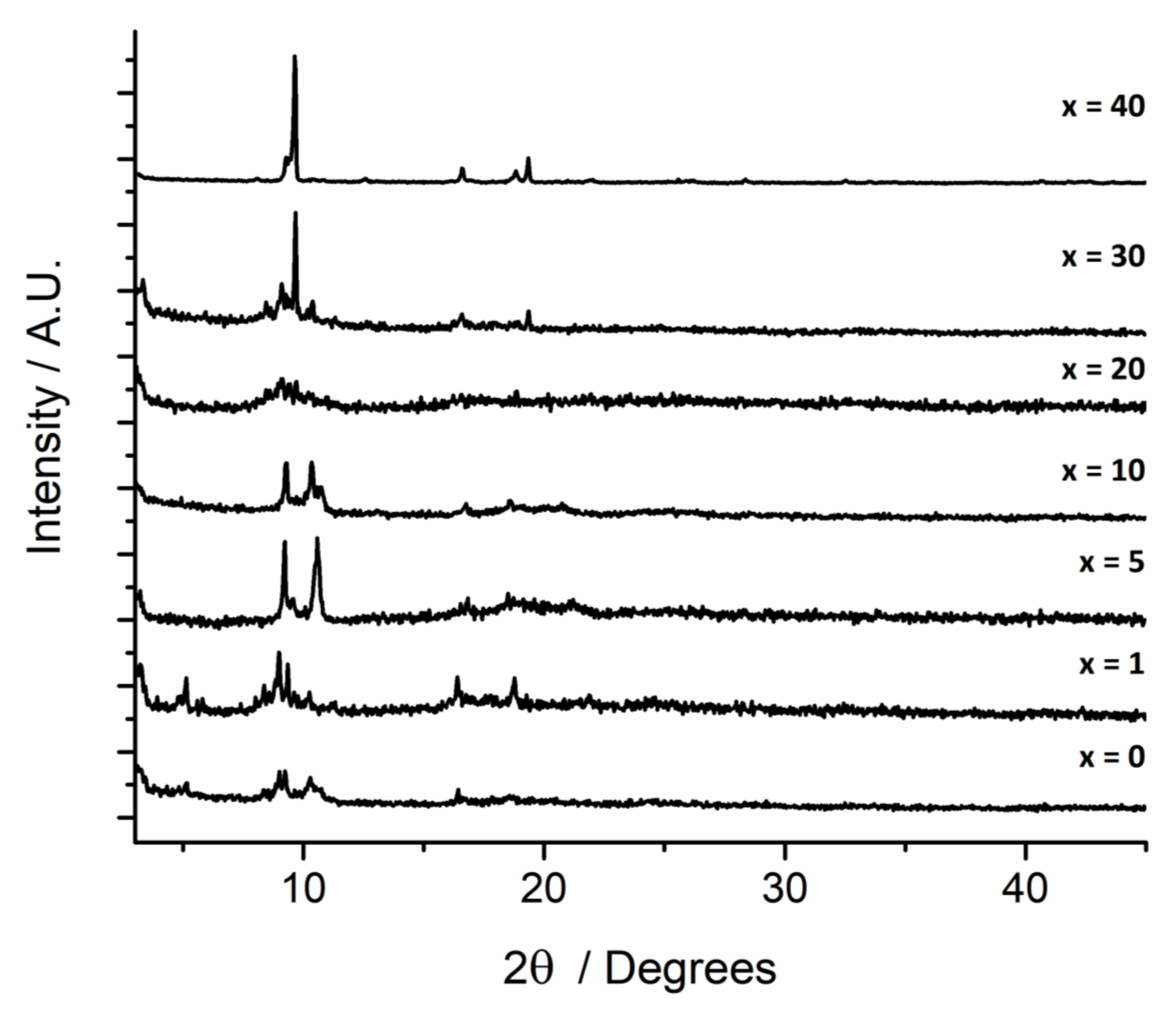

Figure S31. Stacked PXRD patterns of **$Fe(BF_4)_2$-AA**x**(120°C,24h)**, where '**x**' is the number of molar equivalents of acetic acid (**AA**) used in the synthesis.

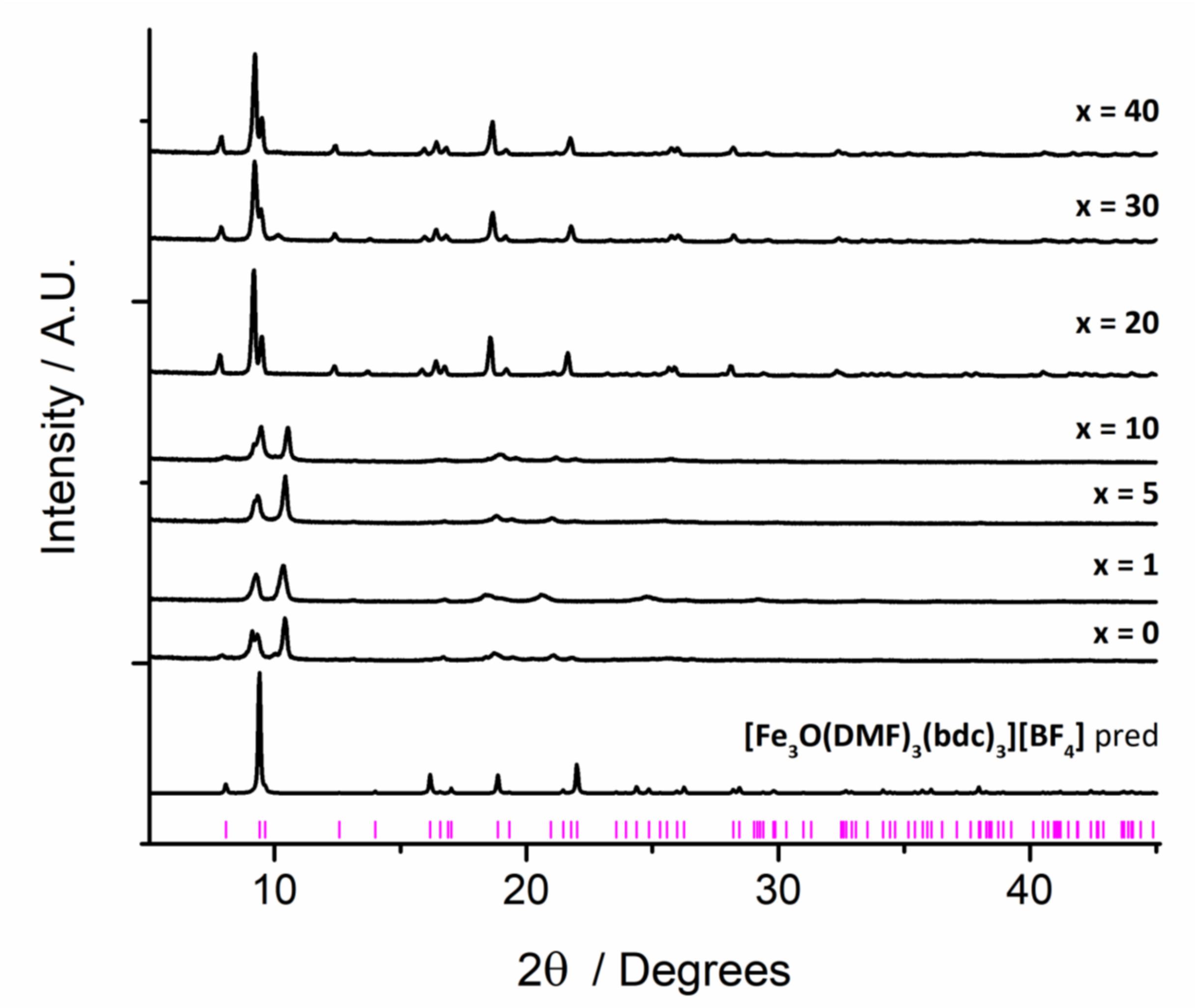

Figure S32. Stacked PXRD patterns of **$Fe(BF_4)_2$-AAx(120°C,72h)**, where '**x**' is the number of molar equivalents of acetic acid (**AA**) used in the synthesis and the predicted pattern of $[Fe_3O(DMF)_3(BDC)_3][BF_4]$ (see Section S5.3) is included for comparison. The patterns where x = 0-10 correspond to that observed for a pristine sample of [Fe(DMF)(BDC)] after washing with DCM and drying (see Section S5.2)

S5.2. Single-Crystal Synthesis of [Fe(DMF)(BDC)]

$Fe(BF_4)_2 \cdot 6H_2O$ (1 mmol) and terephthalic acid (1 mmol) were added to a 50 mL Pyrex reagent jar and DMF (10 mL) was added. The jar was capped and sonicated until the solids dissolved before heating at 150 °C in an isothermal oven. After 3 days, the jar was removed and allowed to cool to room temperature. Yellow polyhedral crystals were evident and the DMF was decanted and replaced several times with fresh DMF, in which the crystals were kept for further analysis.

Crystal data for [Fe(DMF)(BDC)]: $C_{11}H_{11}FeNO_5$, M_r = 293.06, crystal dimensions 0.08 × 0.07 × 0.03 mm, Orthorhombic, a = 19.4334 (19) Å, b = 7.2431 (7) Å, c = 8.7675 (9) Å, V = 1234.1 (2) $Å^3$, T = 150 K, space group *Pnma*, (No. 62), Z = 4, 24397 measured reflections, 1654 independent reflections (R_{int} = 0.033), which were used in all calculations. The final R_1 = 0.032 for 1477 observed data $R[F^2 > 2\sigma(F^2)]$ and $wR(F^2)$ = 0.097 (all data). CCDC Deposition 2088533.

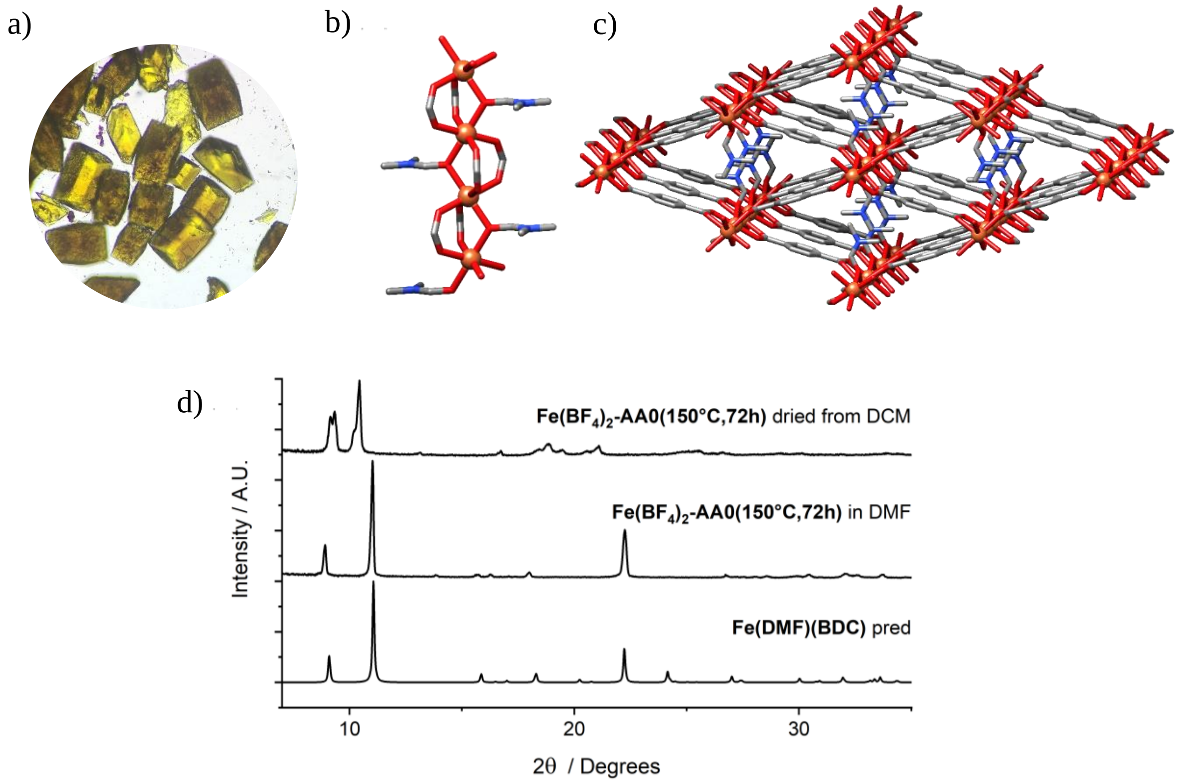

Figure S33.a) Optical image of crystals of [Fe(DMF)(BDC)], b) Chain SBU in [Fe(DMF)(BDC)], c) crystal packing in [Fe(DMF)(BDC)] at a slight angle from the crystallographic *b* axis, d) Comparison of the predicted and experimental PXRD patterns of [Fe(DMF)(BDC)] along with the pattern of the DCM-dried sample, confirming a structural change on drying from DCM and allowing identification in phase experiments.

S5.3. Single-Crystal Synthesis of [$Fe_3O(DMF)_3(BDC)_3$][BF_4]

$Fe(BF_4)_2 \cdot 6H_2O$ (1 mmol) and terephthalic acid (1 mmol) were added to a 50 mL Pyrex reagent jar and DMF (10 mL) was added along with acetic acid (40 mmol). The jar was capped and sonicated until the solids dissolved before heating at 120 °C in an isothermal oven. After 24

hours, the jar was removed and allowed to cool to room temperature. Orange hexagonal-shaped crystals were evident and the DMF was decanted and replaced several times with fresh DMF, in which the crystals were kept for further analysis. Elemental analysis suggests the formula $[Fe_3O(DMF)_3(BDC)_3][BF_4]\cdot1.8H_2O\cdot0.2DMF$. Expected $(Fe_3C_{33}H_{33}O_{16}N_3BF_4\cdot(C_3H_7NO)_{0.2}(H_2O)_{1.8})$: C, 39.22; H, 3.72; N, 4.36. Found: C, 39.21; H, 3.82; N, 4.38.

Crystal data for $[Fe_3O(DMF)_3(BDC)_3][BF_4]$: $C_{33}H_{33}BF_4Fe_3N_3O_{16}$, M_r = 981.98, crystal dimensions 0.12 × 0.1 × 0.01 mm, Hexagonal, $a = b$ = 12.6391 (8), Å, c = 18.3551 (14) Å, V = 2539.3 (4) Å^3, T = 150 K, space group $P\bar{6}2c$, (No. 74), Z = 2, 16518 measured reflections, 2161 independent reflections (R_{int} = 0.038), which were used in all calculations. The final R_1 = 0.055 for 2120 observed data $R[F^2 > 2\sigma(F^2)]$ and $wR(F^2)$ = 0.139 (all data). CCDC Deposition 2088534.

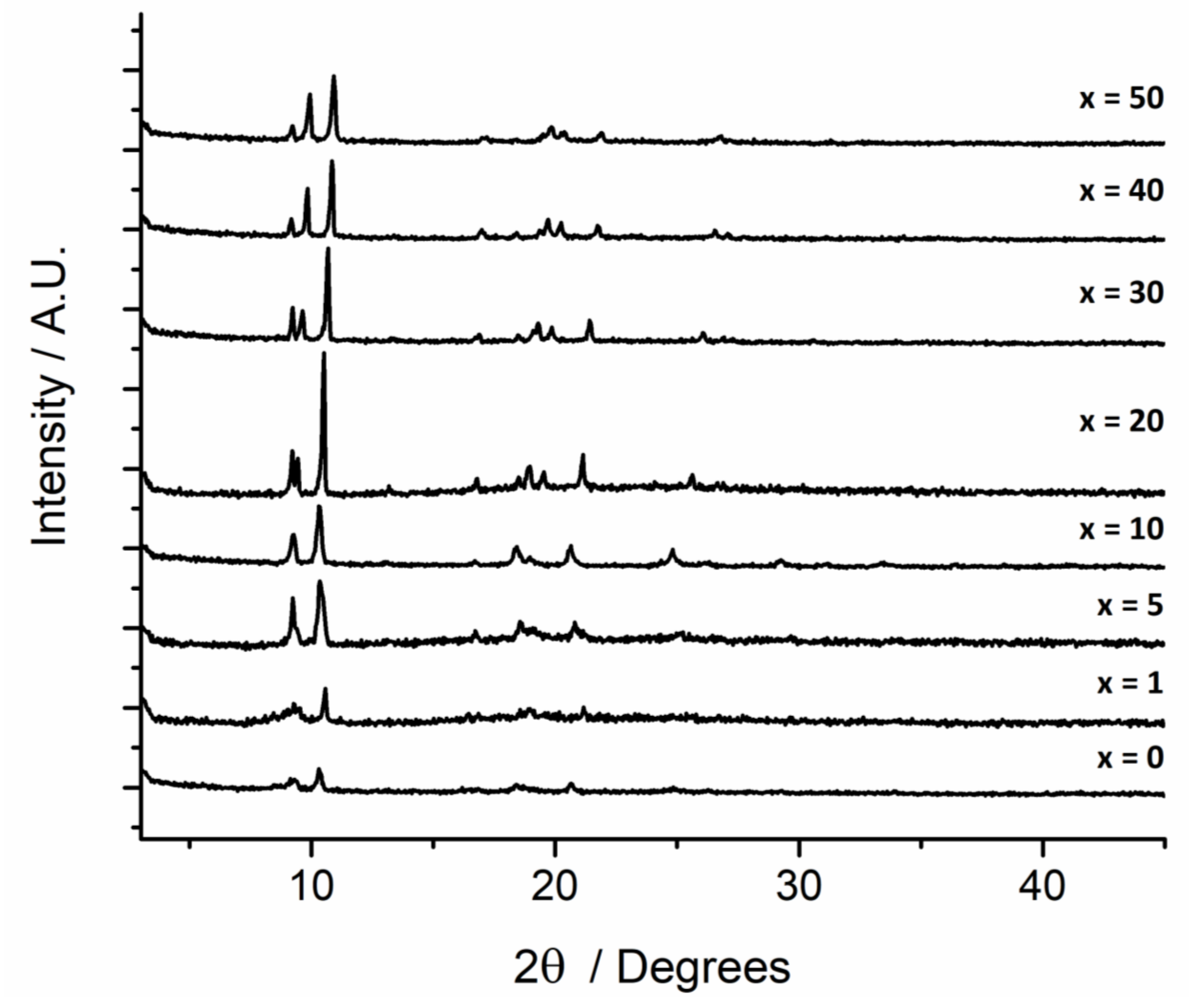

Figure S34. Stacked PXRD patterns of **Fe(OAc)$_2$-AA**x(120°C,24h), where ‘**x**’ is the number of molar equivalents of acetic acid (**AA**) used in the synthesis, showing sole formation of MIL-88B(Fe).

S5.4. Single-Crystal Synthesis of MIL-88B(Fe)

$FeSO_4 \cdot 7H_2O$ (0.05 mmol) and terephthalic acid (0.05 mmol) were added to a 25 mL Pyrex reagent jar and DMF (4 mL) was added along with acetic acid (0.2 mL). The jar was capped and sonicated until the solids dissolved, before heating at 120 °C in an isothermal oven. After 24 hours, the jar was removed and allowed to cool to room temperature. Orange hexagonal rod-shaped crystals were evident and the DMF was decanted and replaced several times with fresh DMF, in which the crystals were kept for further analysis.

Crystal data for MIL-88B(Fe): $C_{24}H_{17}Fe_3O_{16}$, M_r = 728.92, crystal dimensions 0.13 × 0.03 × 0.03 mm, Hexagonal, $a = b$ = 13.9105 (12) Å, c = 17.6608 (13) Å, V = 2959.6 (6) Å^3, T = 150 K, space group $P6_3/mmc$, (No. 194), Z = 2, 32529 measured reflections, 1175 independent reflections (R_{int} = 0.055), which were used in all calculations. The final R_1 = 0.039 for 1056 observed data $R[F^2 > 2\sigma(F^2)]$ and $wR(F^2)$ = 0.124 (all data). CCDC Deposition 2088535.

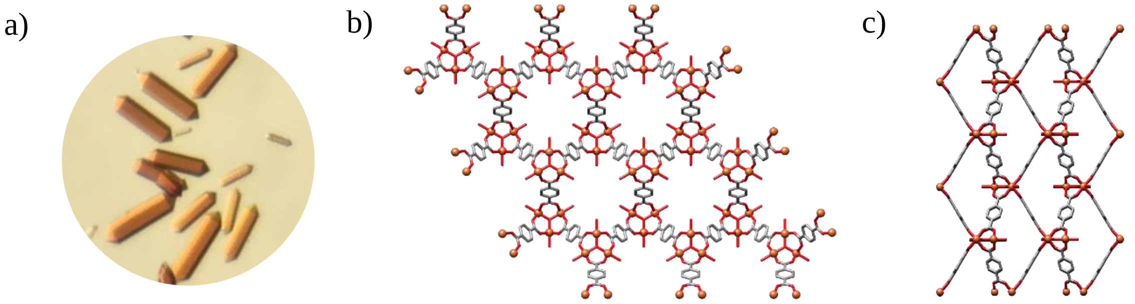

Figure S35. a) Optical image of crystals of MIL-88B(Fe)-DMF, b) packing of MIL-88B(Fe)-DMF as viewed down the *c*-axis, c) packing of MIL-88B(Fe)-DMF as viewed down the *b*-axis.

S5.5. Single-Crystal Synthesis of Fe-BDC-Br

$FeSO_4 \cdot 7H_2O$ (0.05 mmol) and 2-bromoterephthalic acid (0.05 mmol) were added to a 25 mL Pyrex reagent jar and DMF (4 mL) was added along with acetic acid (0.2 mL). The jar was capped and sonicated until the solids dissolved, before heating at 120 °C in an isothermal oven. After 24 hours, the jar was removed and allowed to cool to room temperature. Dark red block-shaped crystals were evident and the DMF was decanted and replaced several times with fresh DMF, in which the crystals were kept for further analysis.

Crystal data for Fe-BDC-Br: $C_{24}H_{15}Br_3Fe_3O_{16}$, M_r = 966.64, crystal dimensions 0.33 × 0.3 × 0.06 mm, Tetragonal, a = 16.3069 (11) Å, b = 16.3069 (11) Å, c = 52.852 (4) Å, V = 14054 (2) Å^3, T = 150 K, space group $I4_1/amd$, (No. 141), Z = 8, 35038 measured reflections, 4705 independent reflections (R_{int} = 0.082), which were used in all calculations. The final R_1 = 0.084 for 3171 observed data $R[F^2 > 2\sigma(F^2)]$ and $wR(F^2)$ = 0.276 (all data). CCDC Deposition 2088537.

S5.6. Bond Valence Sum (BVS) Calculations

Bond valence sum calculations were carried to confirm the oxidation states of the Fe centres in MIL-88B(Fe) and Fe-BDC-Br using the equations given by O'Keeffe *et al*.[S9] This method uses the Fe-O bond lengths around each metal centre to obtain an approximate valence, with an error of about 14%.[S9] The bond lengths, bond valences, and valence sums for each Fe atom are given in Tables S1-3. The BVS equations used are given below:

$$\sum V_{ij} = V_i$$

$$v_{ij} = \exp[(R_{ij}-d_{ij})/b]$$

Where:

v_{ij} is the valence of a bond between 2 atoms i and j

R_{ij} is the valence parameter for a bond between atoms i and j

d_{ij} is the bond length between atoms i and j

b is a universal constant equal to 0.37 Å[S10]

V_i is the valence of an atom i

The R_{ij} value for Fe-O = 1.745 Å[S11]

Table S1. BVS for Fe(1) in **MIL-88B(Fe)**.

**Fe(1)**	Bond length d_{ij} / Å	Bond valence v_{ij}
Fe1—O1	2.013	0.485
Fe1—O1^{i}	2.013	0.485
Fe1—O1^{ii}	2.013	0.485
Fe1—O1^{iii}	2.013	0.485
Fe1—O2	1.9215	0.621
Fe1—O3	2.043	0.447
Sum		V_i =3.006

Symmetry codes: (i) *x*, *y*, -*z*+1/2; (ii) -*x*+*y*+1, *y*, -*z*+1/2; (iii) -*x*+*y*+1, *y*, *z*.

Table S2. BVS for Fe(1) in **Fe-BDC-Br**.

**Fe(1)**	Bond length d_{ij} / Å	Bond valence v_{ij}
Fe1—O1	2	0.502
Fe1—O2^{ii}	2.128	0.355
Fe1—O2	2.128	0.355
Fe1—O2^{iii}	2.128	0.355
Fe1—O2^{i}	2.128	0.355
Fe1—O5	2.09	0.394
Sum		V_i = 2.316

Symmetry codes: (i) *x*, -*y*+1/2, *z*; (ii) -*x*+1, *y*, *z*; (iii) -*x*+1, -*y*+1/2, *z*.

Table S3. BVS for Fe(2) in **Fe-BDC-Br**.

**Fe(2)**	Bond length d_{ij} / Å	Bond valence v_{ij}
Fe2—O1	1.855	0.743
Fe2—O3^{i}	1.991	0.514
Fe2—O3	1.991	0.514
Fe2—O4^{i}	2.028	0.465
Fe2—O4	2.028	0.465
Fe2—O6	2.128	0.355
Sum		V_i = 3.057

Symmetry codes: (i) *x*, -*y*+1/2, *z*.

S.6. DFT Calculations

To understand the relative stability of MIL-88B(Fe) (**acs**) and MIL-88B(Fe) (**snw**), we performed density functional theory (DFT) calculations. To obtain a more accurate description of the Fe^{2+}/Fe^{3+} ions with unpaired electrons, we have used a hybrid DFT functional. All DFT calculations have been performed using the CP2K code (version 7.1), which uses a mixed Gaussian/plane-wave basis set.[S12, S13] We employed double-ζ polarization quality Gaussian basis sets[S14] and a 400 Ry plane-wave cutoff for the auxiliary grid, in conjunction with the Goedecker-Teter-Hutter pseudopotentials.[S15, S16] All DFT calculations were performed in the Γ-point approximation with sufficiently large supercells. Total energy calculations and structural optimizations, including both atomic coordinates and cell parameters, were performed under periodic boundary conditions at the hybrid DFT level using the PBE0 exchange and correlation functional,[S17, S18] which has 25% Hartree-Fock exchange (HFX), with Grimme's D3 van der Waals correction (PBE0+D3).[S19] The HFX calculations were significantly accelerated by using the auxiliary density matrix method (ADMM)[S20] and a truncated Coulomb potential, with which the HFX energy becomes zero beyond a pre-defined real-space cutoff radius. A convergence threshold of 1.0×10^{-6} Hartree was used for the self-consistent field cycle, and structural optimizations were considered to have converged when the maximum force on all atoms falls below 4.5×10^{-4} Hartree/Bohr.

S.7. References

S1. B. H. Toby and R. B. Von Dreele, *J. Appl. Cryst.*, 2013, **46**, 544-549.
S2. T. R. Whitfield, X. Wang, L. Liu and A. J. Jacobson, *Solid State Sci.*, 2005, **7**, 1096-1103.
S3. F. Millange, N. Guillou, R. I. Walton, J.-M. Grenèche, I. Margiolaki and G. Férey, *Chemical Communications*, 2008, 4732-4734.
S4. G. Férey, C. Mellot-Draznieks, C. Serre, F. Millange, J. Dutour, S. Surblé and I. Margiolaki, *Science*, 2005, **309**, 2040-2042.
S5. A. Fateeva, P. Horcajada, T. Devic, C. Serre, J. Marrot, J.-M. Grenèche, M. Morcrette, J.-M. Tarascon, G. Maurin and G. Férey, *Eur. J. Inorg. Chem.*, 2010, **2010**, 3789-3794.
S6. A. C. Sudik, A. P. Côté and O. M. Yaghi, *Inorg. Chem.*, 2005, **44**, 2998-3000.
S7. P. Horcajada, F. Salles, S. Wuttke, T. Devic, D. Heurtaux, G. Maurin, A. Vimont, M. Daturi, O. David, E. Magnier, N. Stock, Y. Filinchuk, D. Popov, C. Riekel, G. Férey and C. Serre, *J. Am. Chem. Soc.*, 2011, **133**, 17839-17847.
S8. K. Karthikeyan, S. Amaresh, S. N. Lee, V. Aravindan and Y. S. Lee, *Chem. Asian J.*, 2014, **9**, 852-857.
S9. N. E. Brese and M. O'Keeffe, *Acta Cryst. B*, 1991, **47**, 192-197.
S10. I. D. Brown and D. Altermatt, *Acta Cryst. B*, 1985, **41**, 244-247.
S11. M. O'Keefe and N. E. Brese, *J. Am. Chem. Soc.*, 1991, **113**, 3226-3229.
S12. J. Hutter, M. Iannuzzi, F. Schiffmann and J. VandeVondele, *Wiley Interdiscp. Rev. Comput. Mol. Sci.*, 2014, **4**, 15-25.
S13. J. VandeVondele, M. Krack, F. Mohamed, M. Parrinello, T. Chassaing and J. Hutter, *Comput. Phys. Commun.*, 2005, **167**, 103-128.
S14. J. VandeVondele and J. Hutter, *J. Chem. Phys.*, 2007, **127**, 114105.
S15. S. Goedecker, M. Teter and J. Hutter, *Phys. Rev. B*, 1996, **54**, 1703-1710.
S16. M. Krack, *Theor. Chem. Acc.*, 2005, **114**, 145-152.
S17. C. Adamo and V. Barone, *J. Chem. Phys.*, 1999, **110**, 6158-6170.
S18. M. Ernzerhof and G. E. Scuseria, *J. Chem. Phys.*, 1999, **110**, 5029-5036.
S19. S. Grimme, J. Antony, S. Ehrlich and H. Krieg, *J. Chem. Phys.*, 2010, **132**, 154104.
S20. M. Guidon, J. Hutter and J. VandeVondele, *J. Chem. Theory Comput.*, 2010, **6**, 2348-2364.
